# Extensive local adaptation within the chemosensory system following *Drosophila melanogaster*'s global expansion

**DOI:** 10.1038/ncomms11855

**Published:** 2016-06-13

**Authors:** J. Roman Arguello, Margarida Cardoso-Moreira, Jennifer K. Grenier, Srikanth Gottipati, Andrew G. Clark, Richard Benton

**Affiliations:** 1 Center for Integrative Genomics, Faculty of Biology and Medicine, University of Lausanne, CH-1015 Lausanne, Switzerland; 2 Department of Molecular Biology and Genetics, Cornell University, Ithaca, New York 14853, USA; 3 Department of Biological Statistics and Computational Biology, Cornell University, Ithaca, New York 14853, USA

## Abstract

How organisms adapt to new environments is of fundamental biological interest, but poorly understood at the genetic level. Chemosensory systems provide attractive models to address this problem, because they lie between external environmental signals and internal physiological responses. To investigate how selection has shaped the well-characterized chemosensory system of *Drosophila melanogaster*, we have analysed genome-wide data from five diverse populations. By couching population genomic analyses of chemosensory protein families within parallel analyses of other large families, we demonstrate that chemosensory proteins are not outliers for adaptive divergence between species. However, chemosensory families often display the strongest genome-wide signals of recent selection within *D. melanogaster*. We show that recent adaptation has operated almost exclusively on standing variation, and that patterns of adaptive mutations predict diverse effects on protein function. Finally, we provide evidence that chemosensory proteins have experienced relaxed constraint, and argue that this has been important for their rapid adaptation over short timescales.

## Abstract (web summary)

Fruit flies gain valuable information about their environment by sensing chemicals. Here, Arguello *et al.* show strong signals of recent selection on the chemosensory system of the fruit fly *Drosophila melanogaster*, consistent with the adaptation of populations to their local chemical environment.

## Graphical abstract

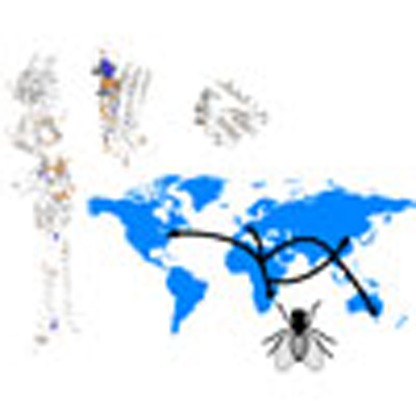

Understanding how organisms adapt to new environments—local adaptation—is of fundamental biological interest. While there is extensive evidence for local adaptation based on phenotypic data, its genetic basis in natural populations is poorly understood^1^. Identifying the precise molecular change(s) that underlie the selected trait(s) remains challenging, as does answering general questions regarding the mutational sources (*de novo*, standing variation) and overall frequency of adaptive evolution^2^,^3^,^4^. Addressing these challenges demands both an in-depth characterization of population genetic variation and a detailed molecular understanding of the biological system under selection.

A particularly interesting question is how neural sensory perception is altered during local adaptation. Sensory systems interact directly with the environment, and are responsible for translating external visual, chemical, mechanical and thermosensory signals into changes in physiology and behaviour. The match between perceptual ability and behavioural outputs carries numerous fitness consequences, for example, the ability to locate food and breeding sites, avoid danger, identify mates and regulate body temperature. Because new environments can present novel stimuli, it is suspected that many sensory systems have experienced strong selective pressures to evolve quickly.

The chemosensory systems of the fruit fly *Drosophila melanogaster*, underlying olfaction and gustation, provide attractive models to address the genetic basis of local adaptation. Laboratory studies have defined many molecular, physiological and anatomical properties of *D. melanogaster*'s chemosensory circuits^5^,^6^,^7^,^8^. In nature, the environmental chemical universe relevant for *D. melanogaster*'s survival is vast, encompassing both volatile and non-volatile signals. These can indicate sources of nutrition, oviposition sites and dangers such as poisonous microbes^9^ and predators^10^, as well as pheromones that control mating, aggression and aggregation behaviours^11^,^12^.

Environmental chemicals are detected in *D. melanogaster* by chemosensory neurons housed within porous cuticular hairs called sensilla^5^. Olfactory sensilla, which detect volatile chemicals, are located on two head appendages, the antenna and maxillary palp. Gustatory sensilla are distributed more widely, on the labellum of the proboscis, leg tarsi, wing margins and, in females, the ovipositor. Chemical detection by these sensory structures requires their direct (or close) contact with a substrate. The *Drosophila* larva also possesses a number of specialized olfactory and gustatory organs^5^.

The vast majority of receptors that detect chemical signals and convert ligand binding into neural activity belong to one of three repertoires, each comprising ∼60 genes: odorant receptors (ORs) and gustatory receptors (GRs), which encode related families of seven transmembrane domain ion channels^5^, and ionotropic receptors (IRs), which are distantly related to ionotropic glutamate receptors (iGluRs)^13^. Olfactory organs express ORs and a subset of IRs (∼15 genes; termed ‘olfactory IRs'^14^), with most olfactory sensory neurons expressing a single ‘tuning' OR or IR that is the principal determinant of the odour-response profile. Gustatory sensory neurons express GRs and the complementary subset of ∼45 ‘non-olfactory' IRs, with individual neurons often expressing multiple GR and/or IR genes^8^,^15^,^16^. In addition to these transmembrane proteins, perireceptor proteins of the odorant-binding protein (OBP) family are secreted into the sensillum lymph that bathes chemosensory neuron dendrites. Despite their name, OBPs (encompassing ∼50 genes) are expressed in both olfactory and gustatory organs, usually in specific subsets of sensilla, where they are thought to contribute to chemosensory signal transduction by solubilizing, transporting and/or protecting chemical ligands from degradation within the aqueous lymph before reaching the sensory membranes^17^.

Previous comparative studies have highlighted the evolution of chemosensory gene families (*Ors*, *Grs*, *Irs* and *Obps*) as ‘dynamic', in terms of high protein divergence, expression differences and family member turnover^15^,^18^,^19^,^20^. Although these changes have occasionally been associated with ecological differences between species^18^,^20^,^21^, very little is currently known about the adaptive (and non-adaptive) function that these changes might have provided. Moreover, almost nothing is known about within-population variability for these families, as most evolutionary investigations have focused on inter-species comparisons. At these deeper timescales (that is, many millions of years), the short-lived DNA-based signals of selection are largely eroded^22^, and the accumulation of non-selected substitutions complicates the identification of the beneficial mutation(s).

To gain a broad understanding of the evolutionary forces governing the *D. melanogaster* chemosensory families and to identify specific targets of selection, we have analysed the genome-wide data (single nucleotide polymorphism (SNP), indel and larger copy number variants (CNVs)) from the recently sequenced global diversity lines (GDLs)^23^. These 84 lines encompass an ancestral-like African *D. melanogaster* population (Zimbabwe) and four derived populations from North America (Ithaca, USA), Europe (Netherlands), Asia (Beijing) and the South Pacific (Tasmania). The African ancestral population of *D. melanogaster* is believed to have expanded ∼60,000 years ago and subsequent lineages have inhabited ecologically diverse localities world wide^24^. These genomic samples are therefore well suited for testing how local adaptation has impacted the chemosensory system, and to provide the first view into how these systems vary among distinct populations.

By placing genomic analyses of chemosensory protein families in the context of those of other large families, we demonstrate that chemosensory proteins as a group do not display exceptional rates of adaptive divergence. By contrast, more recent signals of historical selection arising from within-species analyses reveal striking evidence for selection in chemosensory protein families. Moreover, these analyses indicate that standing variation has provided the primary substrate for selection, and that this variation likely has diverse effects on protein function.

## Results

### Molecular divergence of large protein families

We were interested in quantifying the extent to which chemosensory proteins experience adaptive evolution over short time spans relative to other regions of *D. melanogaster*'s genome. Because the main chemosensory gene families are large (∼60 members each) and often tandemly arranged, we used all other large multigene families (≥20 members) for our standard of comparison. Protein family definitions were based on PANTHER Database classifications^25^, and encompass 40 families (Supplementary Table 1) with known or predicted roles in diverse biological processes such as immune defence and metabolism.

Like chemosensory genes, members of these multigene families are broadly distributed across *D. melanogaster*'s major chromosome arms and recombination environments (Supplementary Fig. 1). In addition, the use of protein families provides a more natural comparison across groups of genes with varying degrees of functional overlap than a random set of loci.

**Figure 1:**
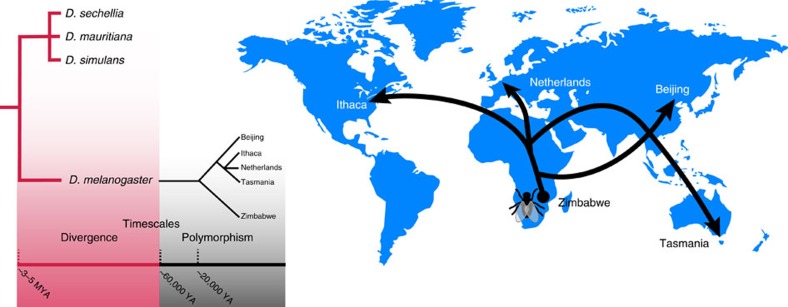
*D. melanogaster*'s recent global expansion. Left: tree schematic illustrating *D. melanogaster*'s relationship with its most closely related species, and the relationship between the five *D. melanogaster* populations from the global diversity lines. The most recent common ancestor shared with the *D. simulans* species trio was ∼3–5 million years ago (MYA). The non-African populations are estimated to have branched off ∼20,000 years ago (YA), and it is believed that the African ancestral population of *D. melanogaster* began to expand ∼60,000 YA. The separation between the two trees (red versus black) emphasizes the two timescales examined in this study. Right: cartoon representation of the expansion of the five populations from Africa around the globe.

Our polymorphism data originated from the GDLs, for which there are validated calls for ∼5.8 million SNPs and 970,000 small indels^23^. In addition, we have incorporated CNV calls consisting of 2,221 duplications, 56,562 deletions and 3,850 insertions^26^. These polymorphism and divergence data allow us to test models of adaptive evolution at two different timescales, and provide information about adaptive changes that occurred as *D. melanogaster* was forming as a species, as well as local adaptation during its recent global expansion (Fig. 1).

### Chemosensory genes are not outliers for adaptive divergence

We first investigated the occurrence of relatively old signals of selection within these large protein families along the branch leading to extant *D. melanogaster* after it split from its last common ancestor with the *D. simulans* triad (∼3–5 Myr ago^27^; Fig. 1). In particular, we tested whether chemosensory genes experienced a disproportionate number of positively selected protein changes along this branch when compared with the other large families. Central to our tests were the numbers of silent and replacement polymorphism (*P*_S_ and *P*_R_, respectively) and silent and replacement substitutions (*D*_S_ and *D*_R_, respectively). These can be compared through contingency tables, referred to as McDonald-Kreitman (MK) tables^28^. If positive selection has acted on protein structures, we would expect a significant excess of replacement changes between species (that is, an excess *D*_R_/*P*_R_ relative to *D*_S_/*P*_s_). Under a neutral model, we would expect equal ratios (*D*_R_/*P*_R_=*D*_s_/*P*_s_). From these data, it is also possible to estimate the fraction of amino-acid differences that were fixed between species by positive selection^2^,^29^.

**Figure 2:**
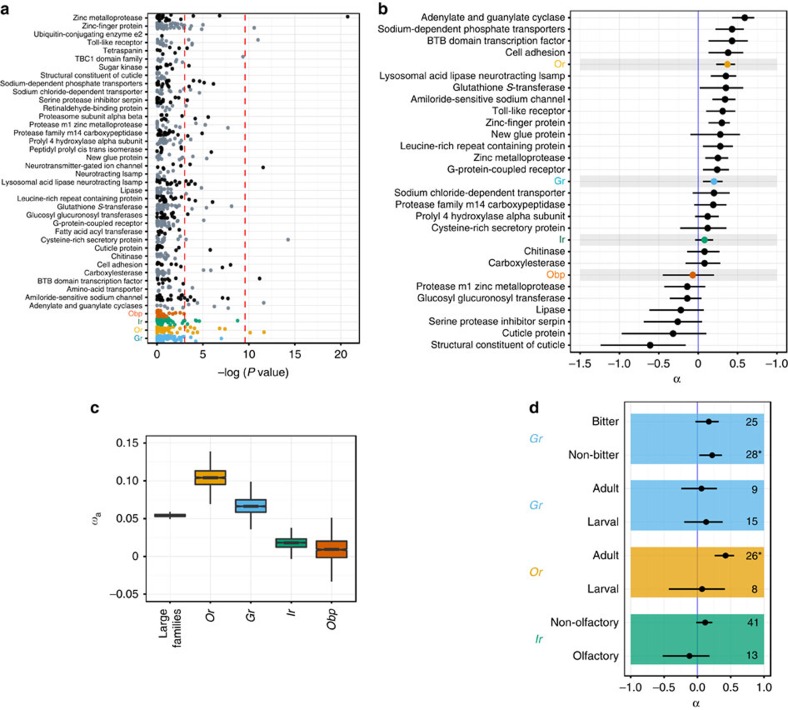
Adaptive divergence analyses. (**a**) Manhattan Plot for large protein family MK test *P* values. Left-most red line denotes the 5% significance level; right-most red line denotes Bonferroni correction significance threshold. (**b**) Rank-ordered plot of the fraction of adaptive substitutions (*α*) inferred across the large protein families. Coloured spheres represent the maximum likelihood *α* estimates, with horizontal lines indicating the 2 units of log(*L*) confidence intervals. The total number of families included in the analyses was reduced to 29 due to data requirements. (**c**) Box plot comparing *ω*_a_ (*α* divided by neutral diversity) among the chemosensory genes and the pooled non-chemosensory large protein families. The boxes show the interquartile range and bars extend to the highest and lowest outliers. (**d**) Comparisons of *α* between functional groupings within gene families. Numbers along the right margin indicate the number of genes included in the analyses, with asterisks indicating significantly positive *α* estimates (*P*<0.05; log likelihood ratio test). Black spheres represent the maximum likelihood *α* estimates, and horizontal lines indicate the 2 units of log(*L*) confidence intervals.

We calculated three related summary statistics based on our MK tables for the 29 large protein families having the most complete data: individual gene MK test *P* values^28^, a summary of the MK tests that controls for sparse data referred to as the direction of selection (DoS^30^), and the fraction of protein changes fixed by positive selection, *α* (refs 2, 29). Although we identified a small number of individual chemosensory genes as potential targets of positive selection (before and after correcting for multiple tests; Fig. 2a; Supplementary Data 1), the chemosensory families do not uniformly have a higher frequency of significant MK tests than the other large protein families nor are their *α* values concentrated in the upper tail (Fig. 2b; similar results were observed for DoS estimates; Supplementary Fig. 2). These data indicate that the chemosensory genes, as a group, are not outliers for having experienced adaptive divergence.

### Adaptive divergence within chemosensory families

Within the four chemosensory families, *Ors* and *Grs* consistently carry the strongest family-wide signals of interspecific adaptive change. Notably, the confidence intervals for the *Or* and *Gr α* estimates are relatively small and do not overlap zero (Fig. 2b); in addition, *Ors* possess the fifth highest estimated *α* value among large protein families. By contrast, the *Irs* and *Obps* provide *α* estimates that are compatible with neutrality (confidence intervals overlap zero; Fig. 2b). A consistent result is obtained if we scale *α* by the rate of synonymous substitution (*ω*_a_), indicating that the observed trend among chemosensory loci, as well as the comparison of chemosensory loci and other large protein families, is not driven by systematic differences in the effectively neutral substitution rates among families^31^ (Fig. 2c).

A set of 17–21 genes (10 *Ors*, 4 *Grs*, 6 *Irs* and 1 *Obp*) shows an excess of nonsynonymous divergence before correcting for multiple tests, depending on whether the substitutions are polarized along *D. melanogaster*'s branch (Supplementary Data 1). One of the *Ors* (*Or67a*) was independently identified as a target of selection within a more limited study of this family^32^. While caution must be applied to this set, as only two are significant after Bonferroni correction (*Or33c* and *Or49a*), several encode receptors with behaviourally relevant ligands. For example, OR49a is narrowly tuned to a *Leptopilina* wasp semiochemical, and is necessary for avoidance of this parasitoid^10^. A second intriguing candidate is GR63a, which is part of a receptor for CO_2_, a potent, but species-specific trigger of avoidance behaviours^33^. These data provide potential inroads for between-species studies of functional differences, to permit rigorous tests of adaptive protein changes.

Within the *Ir* family, five of the six genes that individually carry signals of adaptive evolution are from the non-olfactory subfamily, many of which are expressed in taste organs^8^,^15^. This subfamily has experienced more extensive between-species changes than the olfactory *Irs*, many of which are deeply conserved in insects^15^. We therefore estimated *α* separately for the two *Ir* subfamilies to test whether this accelerated divergence was the result of relaxed constraint or adaptive protein changes. Indeed, the non-olfactory subfamily carries a positive *α* estimate, but its confidence interval does narrowly overlap zero (0.116, −0.01:0.23), while the olfactory subfamily possesses a negative *α* estimate (−0.12, −0.52:0.18; Fig. 2d).

To explore whether other subgroups of chemosensory gene families display different divergence properties, we additionally examined the *Gr* and *Or α* estimates with respect to the subsets of these genes expressed only in the adult or larva, and for the subset of *Gr* genes that encode receptors implicated in bitter tastant detection^5^,^7^ (sample size limits other types of categorization). Adult-specific *Ors* have a significantly positive *α* (0.42, 0.26:0.55; log likelihood ratio test: *P*<0.0001), in contrast to those expressed only in the larva (Fig. 2d). A similar relationship was not observed for the *Gr*s. However, there is an indication that the receptors outside of the bitter clade (including those detecting pheromonal and sweet ligands) are more likely to have experienced adaptive divergence, as their *α* is significantly positive (0.22, 0.03:0.37; log likelihood ratio test: *P*<0.05), while the bitter clade's confidence interval overlaps zero (0.17, −0.03:0.32).

Together, these between-species analyses provide a broader protein family-wide context to interpret chemosensory protein divergence than has previously been available. Importantly, our results suggest that chemosensory families did not contribute disproportionately to adaptive evolution within the ancestral lineage leading to extant *D. melanogaster*. These findings support a more tempered view than has often been taken, in which chemosensory protein families are presented as ‘token' examples of rapid adaptive divergence. Consistent with previous results^2^,^34^, our data indicate that *D. melanogaster*'s protein-coding genome as a whole experienced a large amount of adaptive divergence; chemosensory proteins fit within this greater trend. In this context, our analyses provide novel insights into other large protein families, and highlight those that warrant further investigation for between-species differences. Interestingly, the ‘adenylate and guanylate cyclase' family, which has the highest *α* value (0.59, 0.43:0.71; log likelihood ratio test: *P*<<0.01; Fig. 2b), includes genes implicated in behavioural responses to gustatory stimuli and hypoxia^35^.

### Chemosensory families and rapid local adaptation

We next tested for selective events that have occurred over the past few thousand years within *D. melanogaster* populations. Do similar evolutionary patterns hold across this shallower timescale, and how might the global expansion out of Sub-Saharan Africa into new ecological niches impact the chemosensory protein families?

A common approach to scanning the genome for between-population signals of selection is to test for significant differences in allele frequencies among population samples (*F*_st_-based approaches). Differences in the presence or strength of positive selection across populations can result in changes in allele frequencies, thereby elevating values of *F*_st_. We applied two *F*_st_-based approaches: a Bayesian model-based approach^36^ and a demographically informed empirical-distribution approach.

**Figure 3:**
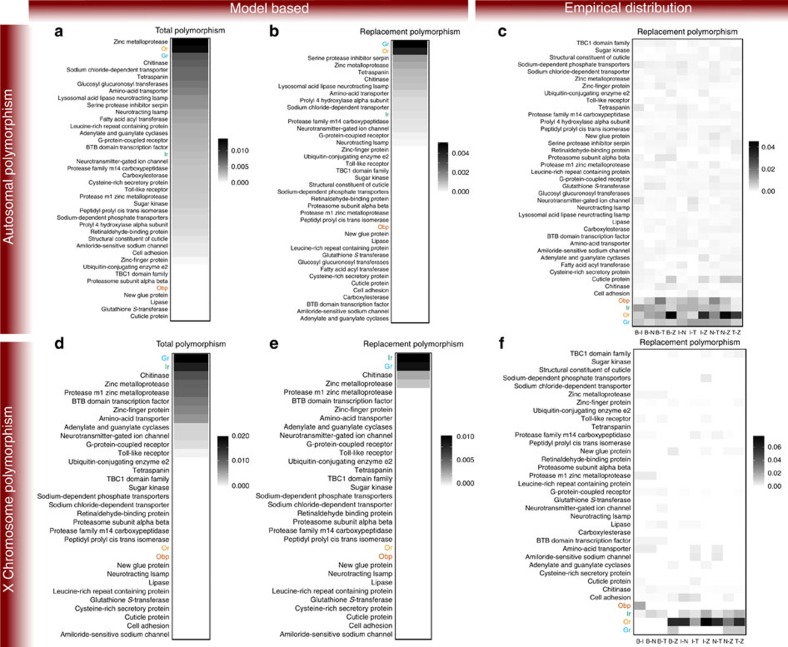
Chemosensory families show strong population differentiation (*F*_st_). Heatplots summarizing the fraction of loci identified as positive selection candidates. The left two columns (**a**,**b**,**d**,**e**) display results from a model-based approach (BayeScan), summarizing either the total data (total polymorphism) or data for replacement polymorphism only (replacement polymorphism). The third column (**c**,**f**) displays results from an empirical-distribution outlier approach, using the 1% *F*_st_ tail as the cutoff. The top row displays results for the autosomal data; the bottom row displays data for the X chromosome. Values shown in the scale bars are the total number of outliers identified in the given analyses divided by the total number of SNPs in the same analysis.

As our initial interest was in the relative rankings among the large protein families, we summarized the results from the Bayesian analysis as the fraction of SNPs identified as outliers, scaled by the total number of SNPs within each family. Due to the varying effective population sizes, these analyses were carried out separately for the autosomes and the X chromosome. The proportion of outlier SNPs for *Ors* and *Grs* on autosomes (0.013 and 0.009, ranking second and third, respectively), and for *Grs* and *Irs* on the X chromosome (0.021 and 0.018, ranking first and second) are among the largest (Fig. 3a,d). When focusing exclusively on protein-changing SNPs, the chemosensory families, except for the *Obps*, rise further in the rankings for the autosomal set (*Grs* are first (0.005), *Ors* are second (0.004) and *Irs* are eleventh (0.0005); Fig. 3b).

These model-based *F*_st_ results are consistent with the contribution of the nonsynonymous *F*_st_ values in the extreme tails of the genome-wide empirical *F*_st_ distributions. If positive selection has operated disproportionally on the sensory protein families, we would expect there to be an enrichment of these genes in the upper tail of the *F*_st_ distribution. We calculated the 1% upper tails from all five pair-wise population nonsynonymous *F*_st_ distributions, and computed the number of nonsynonymous polymorphisms falling within these tails for each of the protein families. We then scaled these counts by the total number of nonsynonymous polymorphisms within each protein family. Notably, the chemosensory genes have a much higher proportion of protein-changing SNPs in the upper tails of the *F*_st_ distribution than most other protein families (Fig. 3c,f). As expected, all loci identified through the Bayesian analysis were identified within the 1% data set.

Our results from examining the empirical distribution of *F*_st_ are robust across both the autosomal and the X-chromosome loci, and are independent of the particular threshold used for identifying the tail (Fig. 3). Furthermore, we used coalescent simulations to explore how likely the observed *F*_st_ values in the extreme tails would be observed under selectively neutral models that include reasonable demographic parameters. Encouragingly, for most pair-wise comparisons, our values demarking the empirical 1 and 5% tails superseded those of the simulations (several Beijing scenarios are exceptions; Supplementary Data 2). These simulation results reinforce the conclusions that the extreme *F*_st_ tails are enriched for targets of positive selection and that chemosensory protein families are among the most quickly adapting proteins in the *D. melanogaster* genome among populations.

### Integrating *F*_st_ outliers with chemosensory protein function

Functional analyses of chemosensory receptors, in particular the ORs, have revealed a range of breadths of tuning profiles, from receptors that respond to only a single compound, to those that detect many chemically diverse molecules^37^,^38^. We asked whether the tuning breadth of the receptors has a relationship with their rate of between-population differentiation. One might suspect, for example, that broadly tuned receptors could more readily be selected upon, as a result of having a larger pool of potential ligands. Conversely, narrowly tuned receptors may be more crucial to the fly's fitness and thus be under stronger purifying selection. We used published receptor specificity data (measured by lifetime kurtosis) for a majority of ORs^39^. We then regressed *F*_st_ values onto these receptor specificity measures. We found a significantly negative correlation between *F*_st_ and the breadth of tuning (−0.24; *P*=0.03), suggesting that broadly tuned receptors differ between *D. melanogaster* populations more than narrowly tuned receptors (Supplementary Fig. 3). Although additional substantiating physiological data are needed, this observation might guide future investigations of the relationship between the specificities of receptors and their rates of evolution.

**Figure 4:**
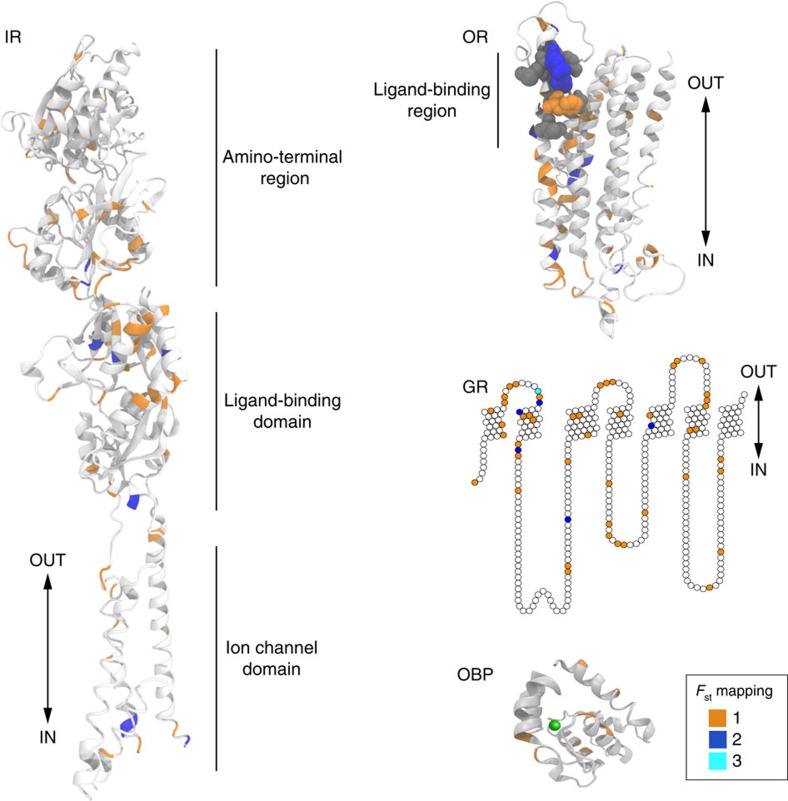
Candidate protein-altering SNPs on chemosensory protein models. Protein model templates for the four chemosensory families onto which residues identified as selection candidates (top 1% *F*_st_) are mapped. The colour coding (bottom right box) indicates the number of times a particular residue was identified as a candidate in pair-wise population comparisons. Although monomeric proteins are shown, IRs and ORs form heteromeric complexes of ligand-tuning receptors with structurally related co-receptors^6^, and GRs are also likely to function in multimeric assemblies^16^. Double-headed arrows next to these protein models indicate the approximate position of the sensory neuron membrane. IR: residues are mapped onto the X-ray crystal structure of the AMPA iGluR (PDB 3KG2). The IR amino-terminal region is much shorter and highly divergent from that of iGluRs, leading to very poor alignment quality, so the precise three-dimensional position of the mapped residues in this region is not informative. OR: residues are mapped onto the OR85b model built by amino-acid coevolutionary and secondary structure analyses^41^. The large dark-grey spheres on the OR structure highlight the location of residues experimentally implicated in influencing ligand recognition properties. We have excluded the high *F*_st_ residues for OR22b and OR22c due to the complex nature of the locus (polymorphic chimeric). GR: residues are mapped onto a snake plot of GR10b, as no three-dimensional information is available. OBP: residues are mapped onto the LUSH (OBP76a) structure (PDB: 2GTE). The green sphere indicates the internal cavity where the ligand is expected to reside.

IRs, ORs and GRs are thought to be ligand-gated ion channels, whose binding of extracellular chemicals induces gating of a transmembrane pore^40^. To investigate whether selection candidate residues cluster within functional domains of these receptors, we mapped the top amino-acid-changing candidate SNPs (1% *F*_st_ outliers) onto reference protein models. The predicted domain organization of IRs is best understood because of their homology to iGluRs, and we found that many of the candidate residues are located within the ligand-binding domain (Fig. 4). However, many also map to the amino-terminal region (which has an important but unclear function in IRs^6^) and the ion-channel domain (Fig. 4). The three-dimensional structure of the heptahelical OR and GR ion channels is unknown, but an OR protein model has been built using amino-acid coevolution patterns and secondary structure predictions^41^. Within this model, many candidate residues map within the N-terminal half of the protein, which is thought to encompass the ligand-binding site (Fig. 4), but others are located more C-terminally (in transmembrane helices and intra- and extracellular loops) where ion conduction may occur^42^ (Fig. 4). A similar distribution was found for candidate sites mapped onto a two-dimensional representation of a GR (Fig. 4). These analyses predict that sites under positive selection can have diverse functional influences on these receptors, including both their ligand-binding and ion conduction properties. For OBPs, sites were mapped onto the X-ray crystal structure of LUSH^43^, revealing their location both in the internal ligand-binding cavity and on the external surface (Fig. 4). This distribution suggests that these sites could have either direct or indirect effects on interactions of these proteins with chemical cues.

### Chemosensory genes carry signatures of selective sweeps

**Figure 5:**
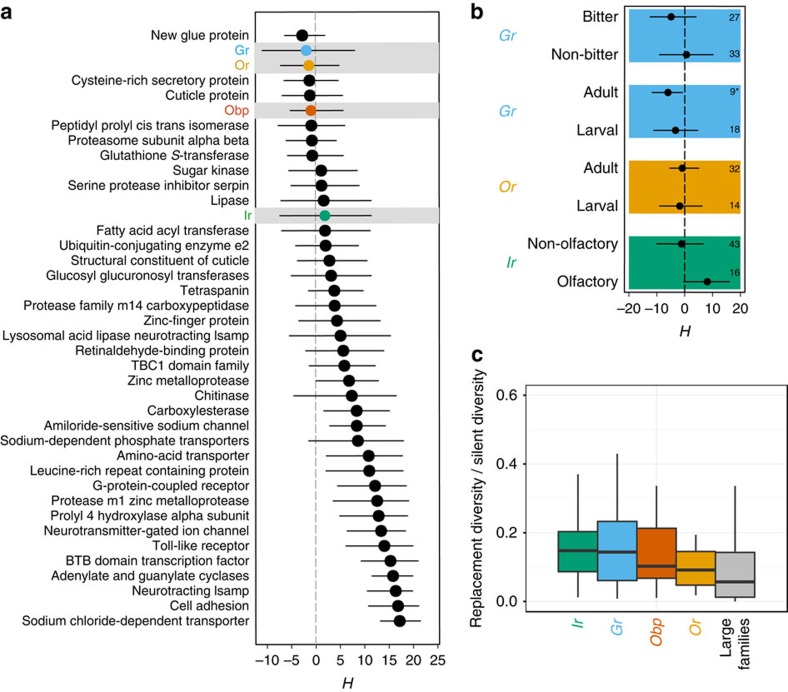
Analyses of nucleotide diversity. (**a**) Rank-ordered distribution of Fay and Wu's *H* values across large protein families. Horizontal lines indicate 95% bootstrap confidence intervals. Negative values indicate an increase in the abundance of high-frequency-derived mutations, which are signals of selective sweeps. Additional coalescent simulations were carried out to test the significance of individual genes (Supplementary Data 3). (**b**) Fay and Wu's *H* values estimated for functional groupings within chemosensory families. Horizontal lines indicate 95% bootstrap confidence intervals. Numbers along the right margin indicate the number of genes included in the analyses, with the asterisk indicating significantly negative *H* (95% confidence interval excludes 0). (**c**) Box plots contrasting the ratio of replacement diversity to silent diversity (a population-level measure of functional constraint) among the chemosensory families and the pooled non-chemosensory protein families. Significant heterogeneity exists among *P*_R_/*P*_S_ values (*P* values<<0.01; Kruskal–Wallis test). The only pair-wise comparisons remaining significant after correcting for multiple tests are the chemosensory versus large family comparisons (Wilcoxon signed-rank tests *P* values<0.005).

Given the striking evidence for positive selection based on allele frequency differences between *D. melanogaster* populations, we reasoned that signatures of selective sweeps might also be borne out in the SNP site frequency spectra (SFS). We tested this hypothesis by computing Fay and Wu's *H* statistic (*H*) across all multigene families^44^; an excess of high-frequency-derived alleles is reflected by negative *H* values and is indicative of a selective sweep. Notably, of the nine families possessing negative *H* estimates, three of these were chemosensory families (*Or*, *Gr* and *Obp*; Fig. 5a). Coalescent simulations, conditioned on the number of segregating sites observed within individual chemosensory genes and over a range of recombination and demographic parameters, identified a number of outliers in all chemosensory families (Supplementary Data 1 and 3). Similar to our divergence analyses (Fig. 2d), we examined the distribution of *H* among stage- and function-specific subgroups of chemosensory families. Here the only functional grouping that alone had a significant signature of adaptation was the adult-specific *Grs* (Fig. 5b).

We additionally carried out a genome-wide selection scan using the composite likelihood ratio (CLR) test^45^. We again observed that chemosensory loci harboured significantly higher CLR values than the other protein families; this suggests that the former harbour a greater proportion of loci that have skews in the SFS, consistent with positive selection (Supplementary Fig. 4). These SFS-based results provide complementary lines of evidence to our *F*_st_ findings, further arguing that sensory protein families are experiencing directional selection at higher rates compared to other large protein families. This unique view of protein family population dynamics highlights the primary role that loci involved in chemosensory perception have had in acting as ‘first responders' when adapting to new ecologies as *D. melanogaster* expanded globally.

Of the proteins that are SFS-based selection candidates, only a few have known ligands, but several of these define sensory pathways linked to specific behavioural phenotypes (Supplementary Data 1). For example, OR47b, OR88a and GR68a are all necessary for the detection of fly-produced chemicals that control different sexual and/or attraction behaviours^46^,^47^, OR49a (introduced above) detects a parasitic wasp semiochemical to mediate avoidance^10^, and GR43a is an internal sensor of fructose involved in feeding regulation^48^. These and other characterized genes represent excellent candidates for future studies linking adaptive mutations to phenotypic consequences.

### Chemosensory families adapt through standing variation

The extent to which adaptive selection acts on standing variation versus *de novo* mutations is a fundamental and debated topic because of its relevance for understanding rates of adaptation^3^,^49^. Having sampled the ancestral-like Zimbabwe population, we were able to address this issue for the *D. melanogaster* chemosensory system. Examination of the set of alleles inferred to be under positive selection (BayeScan based or 1% *F*_st_ tail) indicated that alleles with the derived state regularly segregate in the ancestral range (92%). In addition, most high-frequency-derived mutations within individual genes that carry significantly negative *H* values are variable within the Zimbabwe lines. These data imply that a classic hard sweep model—in which adaptive alleles originate as *de novo* mutations and are quickly fixed—is not supported for the chemosensory loci carrying signals of adaptation.

The observation that selection at chemosensory loci appears to occur rapidly, and predominantly on standing variation, prompted us to seek evidence for divergent selection at different protein-altering positions within the same gene. Instances of this phenomenon would potentially illustrate multiple selective events on the same protein (divergent selection), and may indicate that adaptation at these loci is not mutation limited. To address this question, we investigated genes within this same candidate set (BayeScan based or 1% *F*_st_ tail) that harboured two or more highly differentiated amino-acid-altering polymorphisms between populations. In total, 12 of these genes showed signals of divergent selection between populations for different amino-acid-changing SNPs: *Or22a*, *Or22b*, *Or59a, Gr36b*, *Gr36c*, *Gr59d*, *Gr59e*, *Gr93d*, *Ir11a*, *Ir48b*, *Ir48c* and *Ir75b*. Different populations may therefore have utilized different protein variants from the pool of standing variation to adapt locally.

### Rarity of novel chemosensory genes within *D. melanogaster*

In addition to protein divergence, comparative genomic studies have demonstrated that gene gains and losses are frequent and important events for chemosensory families^50^,^51^. The causes for the changes in family sizes remain unresolved, but have occasionally been correlated with ecology and lifestyles^18^,^20^,^21^.

Using our polymorphism data, where signals of selection and mutational processes remain the strongest, we examined the earliest stages of family size change. For comprehensive quantification of the relative frequencies of functional gains (new gene duplicates) versus functional loss (gene-disrupting mutations), we utilized genome-wide SNP, indel and CNV variant calls^23^,^26^.

Within our set of 2,221 duplications, complete gene duplications of chemosensory loci are rare (4 *Grs* (7%); 5 *Ors* (8%); 0 *Irs* (0%); 2 *Obps* (4%)). Moreover, none of these duplications segregate in >16% of the individuals in one population and only one of the duplications (*Or43b*) segregates in multiple populations (Supplementary Table 2; Supplementary Data 4). These data indicate that recent functional diversification through whole-gene duplication within *D. melanogaster* is rare.

We did uncover, however, several instances of novel chemosensory gene structures resulting from CNVs joining nearby genes. In total, we observe 11 chimeric structures and 6 gene fusions involving chemosensory genes (Supplementary Data 4). While chimeric structures were as likely to involve genes on the same or on opposite strands, all six gene fusions were between chemosensory genes on the same strand. Similar to the whole-gene duplications, nearly all these novel structures are found at low frequencies and/or are unlikely to be functional based on intron/exon structures. The two exceptions are fusions of *Or22a* and *Or22b* (ref. 52) and a novel fusion of *Or65b* and *Or65c* (Supplementary Data 4).

### Polymorphic gene loss is common within chemosensory families

In contrast to the paucity of new genes and protein gene structures, we observed a high frequency of disrupted alleles of chemosensory loci. Among the total set of deletions, sensory genes are significantly overrepresented based on gene ontology functional enrichment tests (Supplementary Data 5; Supplementary Tables 3 and 4). Contrasting the ratios of deletions to duplications, we estimate values ranging from ∼5:1 (*Obp*s, *Gr*s and *Or*s) to 19:0 (*Ir*s). In an evolutionary context, if we assume that the deletions in this set that segregate at >10% are effectively neutral, we would expect drift alone to reduce each of these protein families at 4–14 times the rate that they expand (*Gr*s: 14:0; *Ir*s: 7:0; *Or*s: 6:1; *Obp*s: 4:1). This trend would be consistent with the reduction in the *Or*, *Gr* and *Ir* families that has been inferred using between-species data^15^,^20^.

**Figure 6:**
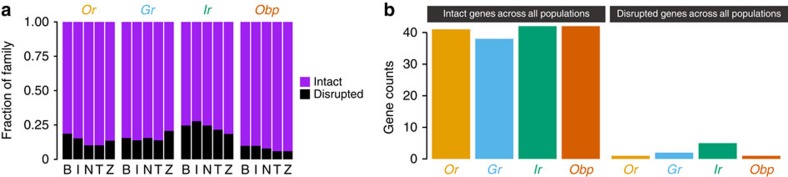
Polymorphic disruptive mutations are common. Summaries for the counts and fraction of the chemosensory families that have null mutations segregating at frequencies ≥10% in the populations. (**a**) The fraction of the gene families that harbour null alleles segregating at ≥10% in the populations. B, Beijing; I, Ithaca; N, Netherlands; T, Tasmania, Z, Zimbabwe. (**b**) Counts of genes that contain either no high-frequency null mutations across the five populations or contain high-frequency mutations in all five populations.

In addition to the CNV data, nonsense mutations within *Gr*s and *Ir*s were roughly twice as frequent compared with other large protein families; a similar trend was not seen for the *Ors* or *Obps* (Supplementary Data 6; Supplementary Table 5). We did not observe any enrichment in small frameshifting indels within the chemosensory loci (Supplementary Table 5). To provide an estimate for the fraction of each of the chemosensory families that harbours loss-of-function mutations, we combined these SNPs and small indels with the CNV disruptions. We additionally required at least one of these disruptive mutations to be segregating ≥10% of the individuals in one population (because mutations were collapsed, there are multiple instances of genes harbouring several null mutations). Summarized in this way, all chemosensory families carry appreciable numbers of null alleles; in some cases, these can be quite high (for example, ∼25% for the *Ir*s) (Fig. 6a). While many chemosensory genes remain intact across all populations, there is a small fraction of each gene family that segregates nulls in all populations (Fig. 6b). Notably, there is no trend with respect to the populations. For example, the Zimbabwe sample does not systematically possess the fewest null alleles, which might have been expected if an out-of-Africa bottleneck was principally responsible for the relaxed constraint in the derived populations.

The mutational target size for gene loss is much larger than for gene gain, and the observed excess of polymorphic disruptive mutations compared with new genes is unsurprising. However, the significant enrichment specifically for chemosensory genes suggests that some are likely to be under relatively relaxed purifying selection, potentially allowing weakly deleterious mutations to persist in the population for longer and at higher frequencies. Overall, selective constraint based on the nucleotide diversity at replacement sites scaled by nucleotide diversity at silent sites (*P*_R_/*P*_S_) indicates that purifying selection is the predominant force acting across all protein families (*P*_R_/*P*_S_<1 for all gene families; Fig. 5c). However, chemosensory genes do have *P*_R_/*P*_S_ distributions that are slightly elevated compared with the background estimate provided by the large protein data set, consistent with weaker purifying selection (Fig. 5c).

## Discussion

The immense molecular and functional diversity of sensory systems between species is increasingly well appreciated. Beyond documenting these differences, however, understanding how such variation emerges within a population, and how it is fixed between species, requires knowledge of the evolutionary forces that govern these changes. Because the genetic signatures required to test models of adaptive evolution are quickly lost^22^, this aim necessitates population genetic data sets.

We have leveraged a population genomic dataset for geographically diverse samples of *D. melanogaster* to investigate the role of adaptive evolution in the recent history of this species' chemosensory system. A striking result that emerged is the contrast between the signatures of adaptive evolution between the divergence (interspecific) and polymorphic (intraspecific) timescales. Chemosensory genes are not outliers for adaptive changes between species in the context of other multigene families. However, within *D. melanogaster*, these genes carry some of the most pronounced signatures of positive selection. Moreover, we have shown that selection has operated predominantly on standing variation, and that there is evidence for multiple advantageous alleles segregating at some loci. In addition, there is strong evidence that the chemosensory protein families are under weaker purifying selection relative to other large protein families, with a higher than expected number of disruptive mutations segregating within them, and elevated *P*_R_/*P*_S_ distributions.

Our detection of signatures of both positive selection and relaxed constraint suggests hypotheses for the modes of evolution experienced by the chemosensory protein families. We propose that chemosensory genes are under weaker purifying selection as a result of: (i) a high level of functional redundancy (overlapping ligand recognition^38^,^53^ or chemosensory-evoked behavioural functions), (ii) fluctuating purifying selection over diverse ecological niches (spatially varying selection) and (iii) a relative freedom from pleiotropic constraints (their action on downstream processes is accomplished solely by the activation of specific classes of chemosensory neurons, and loss of function of these genes does not directly cause lethality or extreme phenotypes). The confluence of these attributes creates a class of genes that would be expected to respond rapidly to selective pressures: there would be ample genetic variation segregating at appreciable frequencies, and little genetic correlation with non-selected traits to impede the direction of selection^3^,^54^. Our demographically diverse *D. melanogaster* samples appear to have provided an opportune timeframe to observe this swift adaptive response to new environments.

Over longer time periods, we propose that signatures of adaptation at other loci ‘catch up' with the initial rapid bout of adaptation of chemosensory genes. This could explain why comparative studies spanning longer time periods would tend to average out selective signals. An additional contributing factor might be that the environmental fluctuations within Africa during the *D. melanogaster* speciation event did not match those that the species endured during its global expansion.

In conclusion, we have shown that the peripheral chemosensory system of *D. melanogaster* shows strong signatures of selection over short timescales. These results, together with the existing and emerging molecular and neurogenetic tools, provide an exciting foundation for investigating the genetics of adaptation at the functional level.

## Methods

### Genomic data

The genomic data used for the study originated from the GDLs^23^, a reference panel consisting of 84 lines derived from five world populations: Beijing–China (15 lines), Ithaca–USA (19 lines), Netherlands (19 lines), Tasmania (18 lines) and Zimbabwe (13 lines)^55^. These lines were inbred for 12 generations and are mostly homozygous, except for regions associated with inversions, which could not be inbred (referred to as ‘heterozygous blocks'^23^). GDLs were fully sequenced to an average depth of 12.5 × per line, and independent validation for both SNP and small indels were generated. These high-quality SNP and small indel calls are publicly available (SRA study SRP050151). In this work, we used the SNP calls that remained after applying the IBD and callability masks^23^. The SNP annotations that were generated by SNPeff^56^ are the same as in the original GDL publication^23^. Nonsense mutations used in the analyses of null alleles were based on these annotations. For the null alleles resulting from small indels, we crossed exon BED files for our gene families with the GDL's small indel VCF file. Frameshifting indels that fell within exon sequences were considered disruptive. SNP diversity estimates per site for our gene sets were generated using vcftools^57^ (v0.1.11). Divergence statistics were based on the available alignment of the GDL SNPs to *D. melanogaster* (dm3), *D. simulans* (droSim2), *D. sechellia* (droSec1), *D. erecta* (droEre2) and *D. yakuba* (droYak2); probabilistic ancestral calls exist for all variable sites. Estimations for the total number of nonsynonymous (ns) and synonymous (s) positions within our gene sets were based on the degeneracy of the codons as annotated by SNPeff: length_ns_=*L*_1_+2/3(*L*_2_)+1/3(*L*_3_) and length_s_=*L*_4_+1/3(*L*_2_)+2/3(*L*_3_), where *L*_*x*_ is the number of *x*-fold degenerate sites.

### CNV data sets

CNVs were identified by integrating the results of three independent CNV detection pipelines: Pindel^58^ (v2.07.11; split-read detection), an in-house pipeline designed around BLAT^59^ (split-read detection) and Delly^60^ (v0.0.7; paired-end detection). The initial set of calls was subjected to several filters and its quality was evaluated by PCR (6–12% false discovery rate depending on whether read depth further supported the call). The final CNV data set consists of 2,221 duplications, 56,562 deletions and 3,850 insertions relative to the reference genome and varying in size between 25 bp and 25 kb (the chosen size limits)^26^. For the gene structure analyses, we defined ‘chimeric' structures as duplication events that partially duplicate two genes to produce a novel gene structure (the original two loci remain unaltered, leading to family size expansion). We defined ‘gene fusions' as genic structures arising by a deletion event that brought together portions of two tandem genes into a single structure (leading to family size reduction).

### Definition of protein families

Protein family groupings were based on the evolutionary and functionally informed classification scheme implemented in the PANTHER database^25^. To extract the large protein families, we downloaded the total set of ‘Protein Classes' from the database. We removed redundant members from these classes and we retained only those families that had ≥20 members. We then cross-referenced the gene IDs within the PANTHER database entries with gene IDs from FlyBase to ensure correct naming convention. Any PANTHER entry that did not identify a gene within FlyBase was removed. We also excluded the chemosensory protein families and replaced them with our own manually curated set. In total, our ‘large protein family' data set (including chemosensory genes) comprises 40 families, encompassing ∼1,200 genes (Supplementary Data 8).

### Polymorphism-divergence tests of selection

Silent and replacement polymorphism was defined by crossing BED files of genes within our large protein family data set with the GDL SNP annotation results^23^ outputted by SNPeff^56^. Divergences were counted based on published probabilistic calls^23^; only positions within the alignments having ≥85% posterior probability were retained. MK based tests^28^ (using a Fisher's exact test) utilized only the African polymorphism data. To avoid tests on genes with too few divergences or too little polymorphism, we required the marginal counts of the MK tables to be >6. We additionally carried out polarized MK tests with these data using the inferred ancestral state calls described above. DoS calculations^30^ were made based on the MK tables using an R script. To estimate the fraction of amino-acid substitutions driven to fixation by positive selection (*α*), and *α* scaled by the synonymous substation rate (*ω*_a_), we used the DoFE package (www.lifesci.susx.ac.uk/home/Adam_Eyre-Walker/Website/Software.html). Divergence, polymorphism, length_ns_ and length_s_ counts that were input for DoFE were calculated using the SNPeff annotations as described above. To create equal sample sizes across the African loci for the *ω*_a_ estimate, we imputed missing data based on the African-specific allele frequencies.

### *F*_st_ analyses

Genome-wide *F*_st_ estimates were generated using the approach of Weir and Cockerham^61^, which allows for unequal sampling between populations. *F*_st_ values for each gene within our large protein family data set were extracted by crossing our BED files with the *F*_st_ files. Similarly, assigning the positions as silent or replacement was achieved with the SNPeff annotations described above.

For input to BayeScan^36^ (v2.1), we filtered all polymorphic sites from our large protein family data set that had a minor allele frequency ≤0.15. We converted our data files from VCF to 012 format using vcftools^57^ (v0.1.12a). We used this resulting 012 file to produce the BayeScan input file using a custom R script. To run BayeScan on each gene family, we modified the default settings so that the ‘-pr_odds' switch was set to 10 and outputted the full trace data.

### SFS scan for selection

We applied the method of Nielsen *et al.*^45^, implemented in SweeD^62^ (v3.2.11), to the full folded SNP data set for each of the five populations independently. For each data set, the CLR was calculated over a grid of 60,000 (-grid 60,000), which resulted in estimates over ∼400 bp. To compare CLRs between gene families (Supplementary Fig. 4), we extracted CLR estimates for each gene family based on the coordinates within the BED files (see above).

### Coalescent simulations

Coalescent simulations to determine outlier *F*_st_ values were carried out using msms^63^.

The topology of the model was based on the previously computed genome-wide *F*_st_^23^, but with a forced polytomy between the short terminal branches of the Netherlands, Ithaca and Tasmania populations. We additionally allowed for migration between the African and ancestral out-of-Africa branch (see Supplementary Fig. 5 and Supplementary Data 8 for the simulation parameters).

The coalescent simulations used to investigate the significance of Fay and Wu's *H* were run using ms^64^. For each chemosensory family, our simulations were based on the median length of the genes (*Or*s and *Grs*=1,500 bp; *Ir*s=2,000 bp; *Obp*s=600 bp). We ran 10,000 simulations for three demographic models (Supplementary Data 8), for three recombination rates (*ρ*=1, *ρ*=50, *ρ*=250), and conditioning on the number of segregating sites within each candidate gene. We calculated summaries of the distribution of Fay and Wu's *H* using the ‘sample_stats' utility within ms^64^. Simulation commands are available in Supplementary Data 7.

### SNP-based summary statistics

Fay and Wu's *H*^44^ was calculated using the ‘stats' utility within the ms distribution^64^. For input into ‘stats', we treated each gene sample as a haplotype by randomly selecting one of two alleles if a given gene contained heterozygous sites. In addition, missing data were imputed based on the population-specific allele frequency of the site.

### Lifetime kurtosis

We obtained lifetime kurtosis (*K*_L_) estimates by first merging available olfactory receptor response data sets within the Database of Odorant Responses^39^ (DoOR). DoOR is an R-based^65^ database, with accompanying data processing functions, and implements a model-based approach for combining heterogeneous receptor response data sets. We used DoOR's ‘modelRP' function to merge data sets where more than one existed for a given receptor. We then estimated the *K*_L_ on this merged response data using formula (1):


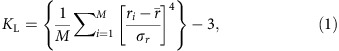


where *M* is the number odorants tested, *r*_*i*_ is the receptor response to the *i*th odorant, 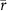 is the overall mean response for the receptor and *σ*_*r*_ is the s.d. of responses the given receptor^66^. To relate *K*_L_ to *F*_st_ estimates, we took the average *F*_st_ across all SNPs within a given receptor's gene, and overall 10 pair-wise population comparisons.

### Mapping residues onto protein models

The most extreme amino-acid-changing SNPs (top 1% *F*_st_ or BayeScan candidates) in chemosensory proteins were mapped onto three-dimensional protein model ‘templates' by generating protein alignments of each family, including the template sequence, using PROMALS3D^67^, locating the equivalent position in the template sequence to each of the candidate selection residues, followed by graphical visualization using VMD^68^. This mapping approach provides a coarse-grained view of the location of candidate selection residues within the proteins, as it is limited by the quality of the alignment of these divergent protein families, and the quality and accuracy of the template structure. For IRs, we aligned all *D. melanogaster* IRs, as well as *D. melanogaster* and selected mammalian iGluRs, and used the X-ray crystal structure of the AMPA family iGluR GluA2 (PDB 3KG2) as a template^69^. For ORs, we used an alignment of *D. melanogaster*, *D. simulans*, *D. sechellia*, *D. erecta* and *D. yakuba* ORs^20^ with the evolutionary coupling-based model of OR85b (version 140_12) as template^41^. For GRs, we used an alignment of *D. melanogaster*, *D. simulans*, *D. sechellia*, *D. erecta* and *D. yakuba* GRs^20^; because neither three-dimensional structure nor models exist, candidate residues were mapped onto a snake plot representation using GR10b as template. For the OBPs, we aligned all drosophilid OBPs^70^ (excluding Obp84a, Obp56c, Obp59b, Obp59a, Obp83ef and Obp83c because of their unusual length), and used the X-ray crystal structure of LUSH as the template (PDB: 2GT3)^43^ within PROMALS3D^67^.

### PCR sequencing

Genomic DNA was extracted by crushing single flies in 50 μl of DNA extraction buffer (10 mM Tris-HCl pH 7.5, 1 mM EDTA, 25 mM NaCl, 200 μg ml^−1^ Proteinase K), incubating for 30 min at 37 °C, before inactivation of Proteinase K with a 5-min incubation at 95 °C. Primers sequences are available in Supplementary Table 6. PCR amplification followed standard protocols followed by Sanger sequencing of the PCR amplicon.

### Data availability

The sequence and annotation data that support the findings of this study have been deposited in NCBI's Sequence Read Archive, with the project identifier SRP050151 (http://www.ncbi.nlm.nih.gov/Traces/sra/sra.cgi?study=SRP050151) (refs 23, 26).

## Additional information

**How to cite this article:** Arguello, J. R. *et al.* Extensive local adaptation within the chemosensory system following *Drosophila melanogaster*'s global expansion. *Nat. Commun.* 7:11855 doi: 10.1038/ncomms11855 (2016).

## Supplementary Material

Supplementary Information Supplementary Figures 1 - 5, Supplementary Tables 1 - 6 and Supplementary References

Supplementary Dataset 1 Summary of selection candidates and functional data. The tables list all protein members within the four main chemosensory families, a subset of functional data ascribed to them, and the selection tests that have identified them as "selection candidates". For the MK-test columns we have separated the tests for which all substitutions between *D. melanogaster* and its last common ancestor have been included (non-polarized), or for only the substitutions along the *D. melanogaster* branch (polarized). We list all genes that have an MK-test p<0.05 with "*" indicating those loci that remain significant after Bonferroni correction. For the two *F*_st_ approaches ("1% *F*_st_ Candidate" and "BayeScan Candidate") we have listed only those genes that contain protein-altering SNPs identified as outliers. For the genes listed in the "Fay and Wu's *H* Candidate" column, we required that a given gene remain significant (p<0.05) across all three of the demographic settings we simulated under, and for at least the two higher recombination rates that examined (ρ = 50 and 250; Supplementary Table 4). Due to the heterogeneity in the methods used to measure tissue expression, we have simplified the classification for this table. In addition to the individual cases cited within the manuscript, we also acquired functional information from published resources.

Supplementary Dataset 2 Fst results based on coalescent simulations under three demographic models. The table provides the empirical 1% threshold for genome-wide *F*_st_ values over all 10 pair-wise population comparisons as well as the 1% and 5% thresholds resulting from the coalescent simulations under three demographic scenarios. The right-most columns indicate whether the empirical thresholds superseded those observed in the simulations.

Supplementary Dataset 3 Coalescent simulations results for Fay and Wu's H under three demographic models and three recombination parameters. The tables provide lists of genes from each of the main chemosensory families inferred to have a negative Fay and Wu's *H* value. Coalescent simulations were carried out using three recombination parameters *(*Rho*)*, and conditioning on the number of segregating sites observed for each gene, with a gene length set to the median gene length for the family (Methods). The right most columns indicate if a given gene's *H* value is significant ("**" indicates significance at the 2.5% level; "*" indicates significance at the 5% level) for the three demographic models examined (Supplementary Fig. 5; Methods). Blank cells indicate that the *H* value was not found to be significant under the given parameters.

Supplementary Dataset 4 Chemosensory CNVs. Detailed information for the chemosensory gene CNVs. Sheet 1 provides the set of coordinates for all CNVs described in the paper (columns A-H), their annotation status details (I-P), their overall frequencies in the total dataset (Q), their presence or absence (1 or 0, respectively) for the CNV within each of the 84 lines (R-DB), whether coverage was used for calling their presence (DC), and their pair-wise population *F*_st_ values. Sheets 2-4 are subsets of sheet 1, to easily access information on the novel gene structures found with the data (gene duplications, chimeric gene structures, and gene fusions).

Supplementary Dataset 5 Population-level summary of duplication and deletion mutations with the chemosensory families. The tables list all members for the four main chemosensory families, color-coded to indicate the presence and frequencies of deletion mutations (color key is in the upper right corner of table). A figure within a cell indicates the number of independent deletions. Bold face gene names indicate that the locus also contains a duplication. The column titled "Shared Between Populations" indicates whether the mutations are also found in other populations. Population abbreviations: B=Beijing; I=Ithaca; N=Netherlands; T=Tasmania; Z=Zimbabwe.

Supplementary Dataset 6 Population-level summary of SNP-based and small indel protein disruptive mutations. The tables list all members for the four main chemosensory families. Color is used to indicate the presence of disruptive mutations given either no frequency cut-off (left tables), or conditional on a 10% frequency cut-off (right tables; color key is in upper right corner of the table). Both SNP and indel mutations are included in the tables, thus some genes harbor more than one class of disruptive mutation. The frequency cut-off was based only on a single class of mutation (we did not sum mutations for the frequency cut-off). Abbreviations for the populations: B=Beijing; I=Ithaca; N=Netherlands; T=Tasmania; Z=Zimbabwe.

Supplementary Dataset 7 Commands for *F_st_* and Fay and Wu's *H simulations*.

Supplementary Dataset 8 Bed files for the protein families used in this study.

## References

[b1] Hereford J. A quantitative survey of local adaptation and fitness trade-offs. Am. Nat. 173, 579–588 (2009). 19272016 10.1086/597611

[b2] Eyre-Walker A. The genomic rate of adaptive evolution. Trends Ecol. Evol. 21, 569–575 (2006). 16820244 10.1016/j.tree.2006.06.015

[b3] Barrett R. D. H. & Schluter D. Adaptation from standing genetic variation. Trends Ecol. Evol. 23, 38–44 (2008). 18006185 10.1016/j.tree.2007.09.008

[b4] Orr H. A. Theories of adaptation: what they do and don't say. Genetica 123, 3–13 (2005). 15881676 10.1007/s10709-004-2702-3

[b5] Vosshall L. B. & Stocker R. F. Molecular architecture of smell and taste in *Drosophila*. Ann. Rev. Neurosci. 30, 505–533 (2007). 17506643 10.1146/annurev.neuro.30.051606.094306

[b6] Abuin L. *et al.* Functional architecture of olfactory ionotropic glutamate receptors. Neuron 69, 44–60 (2011). 21220098 10.1016/j.neuron.2010.11.042 PMC3050028

[b7] Kwon J. Y., Dahanukar A., Weiss L. A. & Carlson J. R. Molecular and cellular organization of the taste system in the *Drosophila* larva. J. Neurosci. 31, 15300–15309 (2011). 22031876 10.1523/JNEUROSCI.3363-11.2011 PMC3225198

[b8] Koh T.-W. *et al.* The *Drosophila* IR20a clade of ionotropic receptors are candidate taste and pheromone receptors. Neuron 83, 850–865 (2014). 25123314 10.1016/j.neuron.2014.07.012 PMC4141888

[b9] Stensmyr M. *et al.* A conserved dedicated olfactory circuit for detecting harmful microbes in *Drosophila*. Cell 151, 1345–1357 (2012). 23217715 10.1016/j.cell.2012.09.046

[b10] Ebrahim S. A. M. *et al.* *Drosophila* avoids parasitoids by sensing their semiochemicals via a dedicated olfactory circuit. PLoS Biol. 13, e1002318 (2015). 26674493 10.1371/journal.pbio.1002318 PMC4687525

[b11] Auer T.O. & Benton R. Sexual circuitry in *Drosophila*. Curr. Opin. Neurobiol. 38, 18–26 (2016). 26851712 10.1016/j.conb.2016.01.004

[b12] Mast J. D. *et al.* Evolved differences in larval social behavior mediated by novel pheromones. eLife 3, e04205 (2014). 25497433 10.7554/eLife.04205 PMC4270068

[b13] Benton R., Vannice K. S., Gomez-Diaz C. & Vosshall L. B. Variant ionotropic glutamate receptors as chemosensory receptors in *Drosophila*. Cell 136, 149–162 (2009). 19135896 10.1016/j.cell.2008.12.001 PMC2709536

[b14] Rytz R., Croset V. & Benton R. Ionotropic receptors (IRs): chemosensory ionotropic glutamate receptors in *Drosophila* and beyond. Insect Biochem. Mol. Biol. 43, 888–897 (2013). 23459169 10.1016/j.ibmb.2013.02.007

[b15] Croset V. *et al.* Ancient protostome origin of chemosensory ionotropic glutamate receptors and the evolution of insect taste and olfaction. PLOS Genet. 6, e1001064 (2010). 20808886 10.1371/journal.pgen.1001064 PMC2924276

[b16] Freeman E. G. & Dahanukar A. Molecular neurobiology of *Drosophila* taste. Curr. Opin. Neurobiol. 34, 140–148 (2015). 26102453 10.1016/j.conb.2015.06.001 PMC4577450

[b17] Leal W. S. Odorant reception in insects: roles of receptors, binding proteins, and degrading enzymes. Annu. Rev. Entomol. 58, 373–391 (2013). 23020622 10.1146/annurev-ento-120811-153635

[b18] Robertson H. & Wanner K. The chemoreceptor superfamily in the honey bee, *Apis mellifera*: expansion of the odorant, but not gustatory, receptor family. Genome Res. 16, 1395 (2006). 17065611 10.1101/gr.5057506 PMC1626641

[b19] Kopp A. *et al.* Evolution of gene expression in the *Drosophila* olfactory system. Mol. Biol. Evol. 25, 1081–1092 (2008). 18296696 10.1093/molbev/msn055 PMC3299402

[b20] McBride C. S. & Arguello J. Roman. Five *Drosophila* genomes reveal nonneutral evolution and the signature of host specialization in the chemoreceptor superfamily. Genetics 177, 1395–1416 (2007). 18039874 10.1534/genetics.107.078683 PMC2147975

[b21] Gilad Y., Wiebe V., Przeworski M., Lancet D. & Pääbo S. Loss of olfactory receptor genes coincides with the acquisition of full trichromatic vision in primates. PLoS Biol. 2, E5 (2004). 14737185 10.1371/journal.pbio.0020005 PMC314465

[b22] Przeworski M. The signature of positive selection at randomly chosen loci. Genetics 160, 1179–1189 (2002). 11901132 10.1093/genetics/160.3.1179 PMC1462030

[b23] Grenier J. K. *et al.* Global diversity lines—a five-continent reference panel of sequenced *Drosophila melanogaster* strains. G3 (Bethesda) 5, 593–603 (2015). 25673134 10.1534/g3.114.015883 PMC4390575

[b24] Stephan W. & Li H. The recent demographic and adaptive history of *Drosophila melanogaster*. Heredity 98, 65–68 (2007). 17006533 10.1038/sj.hdy.6800901

[b25] Mi H., Muruganujan A. & Thomas P. D. PANTHER in 2013: modeling the evolution of gene function, and other gene attributes, in the context of phylogenetic trees. Nucleic Acids Res. 41, D377–D386 (2013). 23193289 10.1093/nar/gks1118 PMC3531194

[b26] Cardoso-Moreira M. *et al.* Evidence for the fixation of gene duplications by positive selection in *Drosophila*. Genome Res. 10.1101/gr.199323.115 (2016). 10.1101/gr.199323.115 PMC4889967 27197209

[b27] Kliman R. M. *et al.* The population genetics of the origin and divergence of the *Drosophila simulans* complex species. Genetics 156, 1913–1931 (2000). 11102384 10.1093/genetics/156.4.1913 PMC1461354

[b28] McDonald J. H. & Kreitman M. Adaptive protein evolution at the *Adh* locus in *Drosophila*. Nature 351, 652–654 (1991). 1904993 10.1038/351652a0

[b29] Bierne N. & Eyre-Walker A. The genomic rate of adaptive amino acid substitution in drosophila. Mol. Biol. Evol. 21, 1350–1360 (2004). 15044594 10.1093/molbev/msh134

[b30] Stoletzki N. & Eyre-Walker A. Estimation of the neutrality index. Mol. Biol. Evol. 28, 63–70 (2011). 20837603 10.1093/molbev/msq249

[b31] Gossmann T. I., Keightley P. D. & Eyre-Walker A. The effect of variation in the effective population size on the rate of adaptive molecular evolution in eukaryotes. Genome Biol. Evol. 4, 658–667 (2012). 22436998 10.1093/gbe/evs027 PMC3381672

[b32] Conceição I. C. & Aguadé M. Odorant receptor (or) genes: polymorphism and divergence in the *D. melanogaster* and *D. pseudoobscura* lineages. PLoS One 5, e13389 (2010). 20967126 10.1371/journal.pone.0013389 PMC2954185

[b33] Jones W. D., Cayirlioglu P., Kadow I. G. & Vosshall L. B. Two chemosensory receptors together mediate carbon dioxide detection in *Drosophila*. Nature 445, 86–90 (2007). 17167414 10.1038/nature05466

[b34] Sella G., Petrov D. A., Przeworski M. & Andolfatto P. Pervasive natural selection in the *Drosophila* genome? PLoS Genet. 5, e1000495 (2009). 19503600 10.1371/journal.pgen.1000495 PMC2684638

[b35] Vermehren-Schmaedick A., Scudder C., Timmermans W. & Morton D. *Drosophila* gustatory preference behaviors require the atypical soluble guanylyl cyclases. J. Comp. Physiol. A Neuroethol. Sens. Neural. Behav. Physiol. 197, 717–727 (2011). 21350862 10.1007/s00359-011-0634-9

[b36] Foll M. & Gaggiotti O. A genome-scan method to identify selected loci appropriate for both dominant and codominant markers: a Bayesian perspective. Genetics 180, 977–993 (2008). 18780740 10.1534/genetics.108.092221 PMC2567396

[b37] Hallem E. & Carlson J. Coding of odors by a receptor repertoire. Cell 125, 143–160 (2006). 16615896 10.1016/j.cell.2006.01.050

[b38] Silbering A. F. *et al.* Complementary function and integrated wiring of the evolutionarily distinct *Drosophila* olfactory subsystems. J. Neurosci. 31, 13357–13375 (2011). 21940430 10.1523/JNEUROSCI.2360-11.2011 PMC6623294

[b39] Galizia C. G., Münch D., Strauch M., Nissler A. & Ma S. Integrating heterogeneous odor response data into a common response model: A door to the complete olfactome. Chem. Senses 35, 551–563 (2010). 20530377 10.1093/chemse/bjq042 PMC2924422

[b40] Silbering A. F. & Benton R. Ionotropic and metabotropic mechanisms in chemoreception: ‘chance or design'? EMBO Rep. 11, 173–179 (2010). 20111052 10.1038/embor.2010.8 PMC2838705

[b41] Hopf T. A. *et al.* Amino acid coevolution reveals three-dimensional structure and functional domains of insect odorant receptors. Nat. Commun. 6, 6077 (2015). 25584517 10.1038/ncomms7077 PMC4364406

[b42] Nakagawa T., Pellegrino M., Sato K., Vosshall L. B. & Touhara K. Amino acid residues contributing to function of the heteromeric insect olfactory receptor complex. PLoS One 7, e32372 (2012). 22403649 10.1371/journal.pone.0032372 PMC3293798

[b43] Kruse S. W., Zhao R., Smith D. P. & Jones D. N. M. Structure of a specific alcohol binding site defined by the odorant binding protein LUSH from *Drosophila melanogaster*. Nat. Struct. Mol. Biol. 10, 694–700 (2003). 10.1038/nsb960 PMC4397894 12881720

[b44] Fay J. C. & Wu C.-I. Hitchhiking under positive darwinian selection. Genetics 155, 1405–1413 (2000). 10880498 10.1093/genetics/155.3.1405 PMC1461156

[b45] Nielsen R. *et al.* Genomic scans for selective sweeps using SNP data. Genome Res. 15, 1566–1575 (2005). 16251466 10.1101/gr.4252305 PMC1310644

[b46] Shankar S. *et al.* The neuropeptide tachykinin is essential for pheromone detection in a gustatory neural circuit. eLife 4, e06914 (2015). 26083710 10.7554/eLife.06914 PMC4491540

[b47] Dweck H. K. M. *et al.* Pheromones mediating copulation and attraction in drosophila. Proc. Natl Acad. Sci. USA 112, E2829–E2835 (2015). 25964351 10.1073/pnas.1504527112 PMC4450379

[b48] Freeman E. G., Wisotsky Z. & Dahanukar A. Detection of sweet tastants by a conserved group of insect gustatory receptors. Proc. Natl Acad. Sci. USA 111, 1598–1603 (2014). 24474785 10.1073/pnas.1311724111 PMC3910600

[b49] Jensen J. D. On the unfounded enthusiasm for soft selective sweeps. Nat. Commun. 5, 5281 (2014). 25345443 10.1038/ncomms6281

[b50] Smadja C., Shi P., Butlin R. K. & Robertson H. M. Large gene family expansions and adaptive evolution for odorant and gustatory receptors in the pea aphid *Acyrthosiphon pisum*. Mol. Biol. Evol. 26, 2073–2086 (2009). 19542205 10.1093/molbev/msp116

[b51] Young J. M. *et al.* Extensive copy-number variation of the human olfactory receptor gene family. The American Journal of Human Genetics 83, 228–242 (2008). 18674749 10.1016/j.ajhg.2008.07.005 PMC2495065

[b52] Aguadé M. Nucleotide and copy-number polymorphism at the odorant receptor genes *Or22a* and *Or22b* in *Drosophila melanogaster*. Mol. Biol. Evol. 26, 61–70 (2009). 18922763 10.1093/molbev/msn227

[b53] Hallem E. A., Dahanukar A. & Carlson J. R. Insect odor and taste receptors. Annu. Rev. Entomol. 51, 113–135 (2006). 16332206 10.1146/annurev.ento.51.051705.113646

[b54] Otto S. P. Two steps forward, one step back: the pleiotropic effects of favoured alleles. Proc. Biol. Sci. 271, 705–714 (2004). 15209104 10.1098/rspb.2003.2635 PMC1691650

[b55] Greenberg A. J., Hackett S. R., Harshman L. G. & Clark A. G. A hierarchical bayesian model for a novel sparse partial diallel crossing design. Genetics 185, 361–373 (2010). 20157001 10.1534/genetics.110.115055 PMC2870970

[b56] Cingolani P. *et al.* A program for annotating and predicting the effects of single nucleotide polymorphisms, snpeff: SNPs in the genome of *Drosophila melanogaster*. Fly 6, 80–92 (2012). 22728672 10.4161/fly.19695 PMC3679285

[b57] Danecek P. *et al.* The variant call format and vcftools. Bioinformatics 27, 2156–2158 (2011). 21653522 10.1093/bioinformatics/btr330 PMC3137218

[b58] Ye K., Schulz M. H., Long Q., Apweiler R. & Ning Z. Pindel: a pattern growth approach to detect break points of large deletions and medium sized insertions from paired-end short reads. Bioinformatics 25, 2865–2871 (2009). 19561018 10.1093/bioinformatics/btp394 PMC2781750

[b59] Cardoso-Moreira M., Arguello J. & Clark A. Mutation spectrum of *Drosophila* CNVs revealed by breakpoint sequencing. Genome Biol. 13, R119 (2012). 23259534 10.1186/gb-2012-13-12-r119 PMC4056370

[b60] Rausch T. *et al.* Delly: structural variant discovery by integrated paired-end and split-read analysis. Bioinformatics 28, i333–i339 (2012). 22962449 10.1093/bioinformatics/bts378 PMC3436805

[b61] Weir B. S. & Cockerham C. C. Estimating f-statistics for the analysis of population structure. Evolution 38, 1358–1370 (1984). 10.1111/j.1558-5646.1984.tb05657.x 28563791

[b62] Pavlidis P., Živkovic D., Stamatakis A. & Alachiotis N. Sweed: likelihood-based detection of selective sweeps in thousands of genomes. Mol. Biol. Evol. 30, 2224–2234 (2013). 23777627 10.1093/molbev/mst112 PMC3748355

[b63] Ewing G. & Hermisson J. Msms: a coalescent simulation program including recombination, demographic structure and selection at a single locus. Bioinformatics 26, 2064–2065 (2010). 20591904 10.1093/bioinformatics/btq322 PMC2916717

[b64] Hudson R. Generating samples under a Wright-Fisher neutral model of genetic variation. Bioinformatics 18, 337–338 (2002). 11847089 10.1093/bioinformatics/18.2.337

[b65] R. Core Team. *A Language and Environment for Statistical Computing*. R Foundation for Statistical Computing, Vienna, Austria. Available at www.R-project.org/ (2015).

[b66] Willmore B. & Tolhurst D. J. Characterizing the sparseness of neural codes. Network 12, 255–270 (2001). 11563529

[b67] Pei J., Kim B.-H. & Grishin N. V. Promals3d: a tool for multiple sequence and structure alignment. Nucleic Acids Res. 36, 2295–2300 (2008). 18287115 10.1093/nar/gkn072 PMC2367709

[b68] Humphrey W., Dalke A. & Schulten K. VMD: Visual molecular dynamics. J. Mol. Graph. 14, 33–38 (1996). 8744570 10.1016/0263-7855(96)00018-5

[b69] Sobolevsky A. I., Rosconi M. P. & Gouaux E. X-ray structure, symmetry and mechanism of an AMPA-subtype glutamate receptor. Nature 462, 745–756 (2009). 19946266 10.1038/nature08624 PMC2861655

[b70] *Drosophila* 12 Genomes Consortium. Evolution of genes and genomes on the *Drosophila* phylogeny. Nature 450, 203–218 (2007). 17994087 10.1038/nature06341

