# Supplementary material for: Extensive local adaptation within the chemosensory system following Drosophila melanogaster's global expansion
Source: Nat Commun. 2016 Jun 13;7:ncomms11855. doi: 10.1038/ncomms11855 (PMC4910016; doi:10.1038/ncomms11855)
Supplement: Supplementary Information — Supplementary Figures 1 - 5, Supplementary Tables 1 - 6 and Supplementary References [file ncomms11855-s1.pdf]

Supplementary Figures

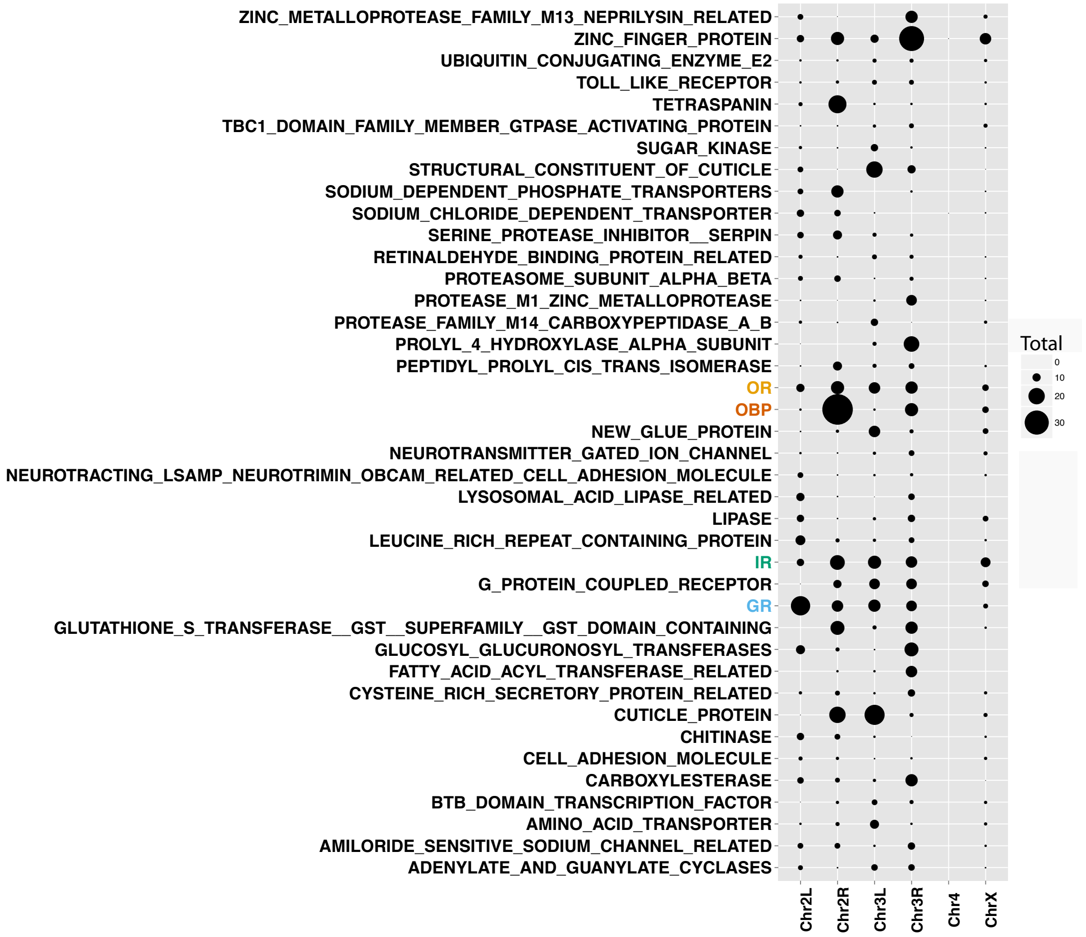

Supplementary Figure 1 | Genomic distribution of large protein families. Chromosomal distribution of the protein families analyzed in this study. Chemosensory families are highlighted in color. Protein definitions for the non-chemosensory protein families are from the PANTHER[1] Database. All major chromosome arms are represented; no family members reside on the small, lowly-recombining fourth chromosome.

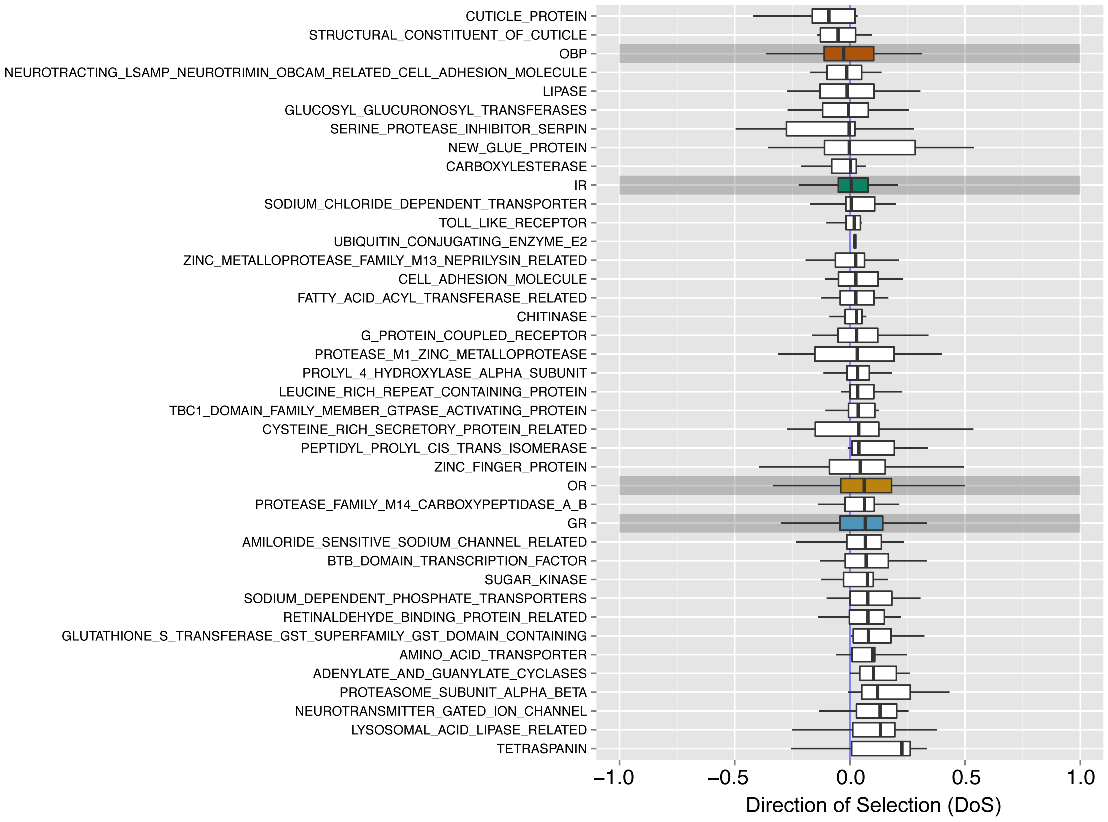

Supplementary Figure 2 | Direction of Selection analysis.

Boxplots for the Direction of Selection (DoS) inferred over the large protein families. Protein families are ordered by their median DoS values. Horizontal lines indicate 95% confidence intervals. Chemosensory families are highlighted in color; all other protein families are plotted as white. In the presence of positive selection, DoS values are positive; values not different from zero are consistent with neutral divergence.

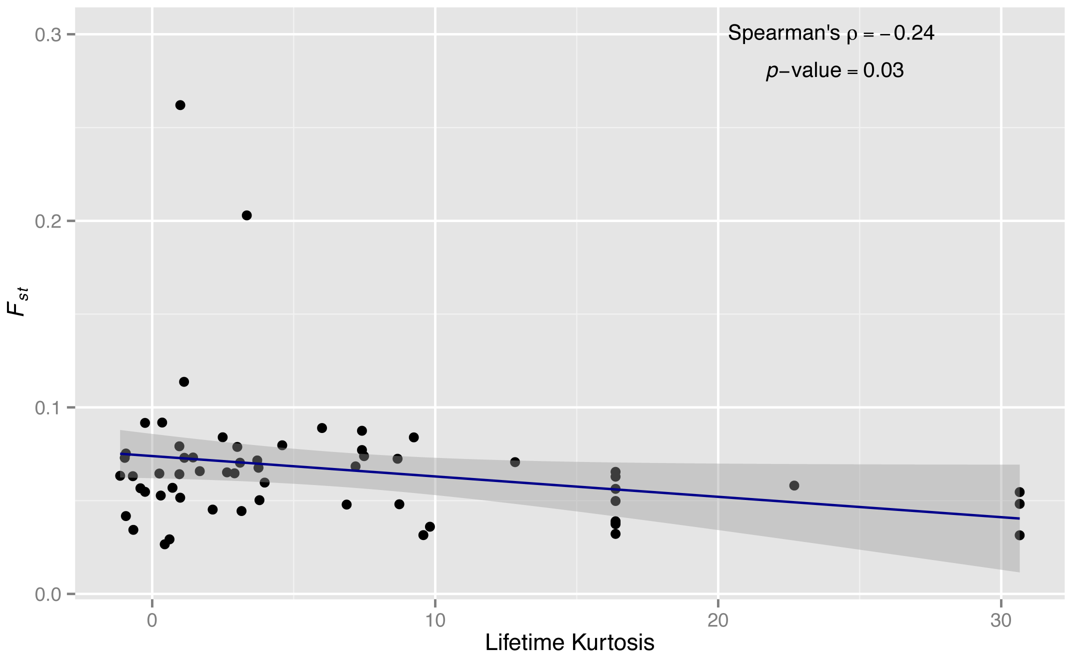

Supplementary Figure 3 | Relationship between OR ligand tuning and population differentiation.

Regression of gene-averaged F_{st} values onto the OR lifetime kurtosis estimates (a measure of receptor tuning breadth; higher values indicate narrower tuning). Lifetime kurtosis values were obtained using data from the DoOR database[2]. F_{st} values were averaged across all ten pairwise population comparisons.

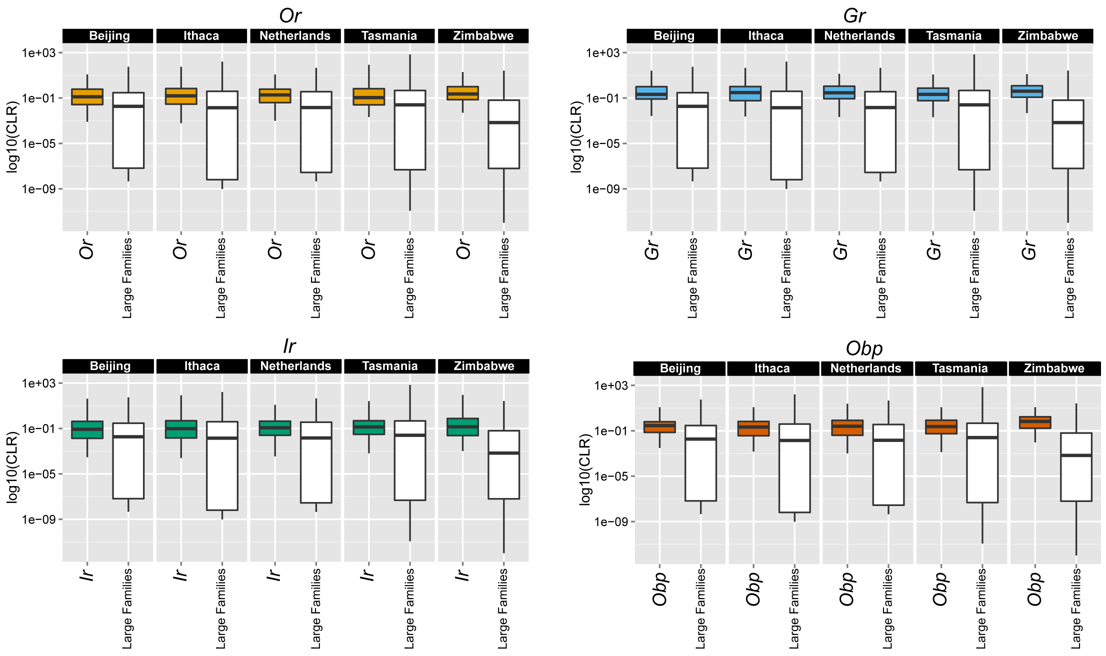

Supplementary Figure 4 | Genomic scan for selection.

Boxplots summarizing scores for a genome-wide scan for selection based on the composite likelihood ratio (CLR) score. Comparisons of the scores between the chemosensory families and pooled non-chemosensory protein families are shown. For all populations, the CLR distributions for chemosensory families are greater than for the other protein families (all Wilcoxon test's *p*-values <<0.05).

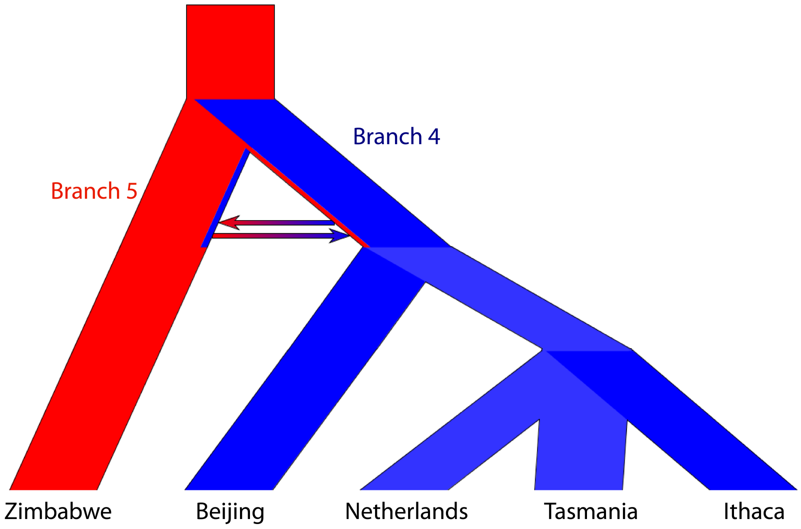

Supplementary Figure 5 | Cartoon representation of the demographic models.

The tree displays the inferred relationship between the populations as inferred by genome-wide F_{st}[3], with the modification that the short terminal branches of Netherlands, Tasmania, and Ithaca populations are forced into a polytomy. This topology was used for the coalescent simulations. The three demographic scenarios (Supplementary Data 5) involve variations in the migration patterns between branches 4 and 5, illustrated with arrows. Model 1 involves asymmetric migration from branch 5 to branch 4; Model 2 involves asymmetric migration from branch 4 to branch 5; Model 3 involves symmetric migration between branches 4 and 5.

Supplementary Tables

PANTHER Names	Shortened Names
ADENYLATE AND GUANYLATE CYCLASES	adenylate and guanylate cyclases
AMILORIDE SENSITIVE SODIUM CHANNEL RELATED	amiloride sensitive sodium channel
AMINO ACID TRANSPORTER	amino acid transporter
BTB DOMAIN TRANSCRIPTION FACTOR	BTB domain transcription factor
CARBOXYLESTERASE	carboxylesterase
CELL ADHESION MOLECULE	cell adhesion
CHITINASE	chitinase
CUTICLE PROTEIN	cuticle protein
CYSTEINE RICH SECRETORY PROTEIN RELATED	cysteine rich secretory protein
FATTY ACID ACYL TRANSFERASE RELATED	fatty acid acyl transferase
GLUCOSYL GLUCURONOSYL TRANSFERASES	glucosyl glucuronosyl transferases
GLUTATHIONE S TRANSFERASE GST SUPERFAMILY GST DOMAIN CONTAINING	glutathione s transferase
G PROTEIN COUPLED RECEPTOR	G protein coupled receptor
LEUCINE RICH REPEAT CONTAINING PROTEIN	leucine rich repeat containing protein
LIPASE	lipase
LYSOSOMAL ACID LIPASE RELATED NEUROTRACTING LSAMP NEUROTRIMIN OBCAM RELATED CELL ADHESION MOLECULE	lysosomal acid lipase neurotracting lsamp
NEUROTRACTING_LSAMP_NEUROTRIMIN_OBCAM_RELATED_CELL_ADHESION_MOLECULE	neurotracting lsamp
NEUROTRANSMITTER GATED ION CHANNEL	neurotransmitter gated ion channel
NEW GLUE PROTEIN	new glue protein
PEPTIDYL PROLYL CIS TRANS ISOMERASE	peptidyl prolyl cis trans isomerase
PROLYL 4 HYDROXYLASE ALPHA SUBUNIT	prolyl 4 hydroxylase alpha subunit
PROTEASE FAMILY M14 CARBOXYPEPTIDASE A B	protease family m14 carboxypeptidase
PROTEASE M1 ZINC METALLOPROTEASE	protease m1 zinc metalloprotease
PROTEASOME SUBUNIT ALPHA BETA	proteasome subunit alpha beta
RETINALDEHYDE BINDING PROTEIN RELATED	retinaldehyde binding protein
SERINE PROTEASE INHIBITOR SERPIN	serine protease inhibitor serpin
SODIUM CHLORIDE DEPENDENT TRANSPORTER	sodium chloride dependent transporter
SODIUM DEPENDENT PHOSPHATE TRANSPORTERS	sodium dependent phosphate transporters
STRUCTURAL CONTITUENT OF CUTICLE	structural contituent of cuticle
SUGAR KINASE	sugar kinase
TBC1 DOMAIN FAMILY MEMBER GTPASE ACTIVATING PROTEIN	TBC1 domain family
TETRASPANIN	tetraspanin
TOLL LIKE RECEPTOR	toll like receptor
UBIQUITIN CONJUGATING ENZYME E2	ubiquitin conjugating enzyme e2
ZINC FINGER PROTEIN	zinc finger protein
ZINC METALLOPROTEASE FAMILY M13 NEPRILYSIN RELATED	zinc metalloprotease

Supplementary Table 1 | PANTHER protein family nomenclature.

Long PANTHER[1] family names and corresponding short versions used for plotting.

Family	Number genes	All duplicated	% gene duplication	Duplicated >= 2 lines per pop	% gene duplication > 10%	coding deletions	% coding deletions	coding deletions >=2 lines per pop	% coding deletion > 10%
Grs	58	4	7%	0	0	23	40%	14	24%
Irs	65	0	0%	0	0%	19	29%	7	12%
Obps	51	2	4%	1	2%	10	20%	4	8%
Ors	59	5	8%	2	3%	24	41%	12	20%

Supplementary Table 2 | Summary of duplication and deletions within chemosensory genes.

The table summarizes the data for duplications and deletions with the main chemosensory gene families in both raw counts as a fraction of the family size. The summaries are also displayed conditioning on the duplication or deletion segregating at >10% in for a given population (count ≥2).

GO term	GO ID	P-value (Holm-Bonferroni correction)	Matches (# of genes)
sensory perception	GO:0007600	6.16E-13	84
sensory perception of chemical stimulus	GO:0007606	7.16E-13	70
neurological system process	GO:0050877	1.83E-08	96
system process	GO:0003008	7.07E-08	103
sensory perception of taste	GO:0050909	2.37E-07	28
detection of chemical stimulus	GO:0009593	2.88E-07	39
proteolysis	GO:0006508	8.01E-04	113

Supplementary Table 3 | CNV-based gene ontology (GO) enrichment.

The significant CNV-based GO enrichment results for all GO terms (“GO ID”), and the number of genes falling within the GO terms (“Matches”).

Protein domain	P-value (Holm-Bonferroni correction)	Matches (# of genes)
7TM chemoreceptor	5.48E-08	25
Protein of unknown function DUF1091	1.59E-07	35
Olfactory receptor, *Drosophila*	1.57E-05	24
Peptidase S1	1.87E-05	59
Trypsin-like cysteine/serine peptidase domain	3.50E-05	59
CHK kinase-like	7.40E-04	20
Cytochrome P450	9.03E-04	27

Supplementary Table 4 | CNV-based protein domain enrichment.

Significant CNV-based protein domain enrichment results, and the number of genes falling within the domain categories (“Matches”).

SNPs			
Family	# Proteins	Nulls	% Nulls
GR	60	22	0.367
IR	61	25	0.410
OR	59	10	0.169
OBP	51	10	0.196
Large_Protein_Families	1060	146	0.138

SNPs			
Family	# Proteins	Nulls >10%	% Nulls
GR	60	8	0.133
IR	61	7	0.117
OR	59	4	0.068
OBP	51	3	0.059
Large_Protein_Families	1060	61	0.058

Small Indels			
Family	# Proteins	Nulls	% Nulls
GR	60	27	0.450
IR	61	39	0.639
OR	59	25	0.424
OBP	51	9	0.176
Large_Protein_Families	1060	435	0.410

Small Indels			
Family	# Proteins	Nulls >10%	% Nulls
GR	60	10	0.167
IR	61	19	0.117
OR	59	7	0.119
OBP	51	5	0.098
Large_Protein_Families	1060	136	0.128

CNVs (all - not restricted to homozygous calls)			
Family	# Proteins	Nulls	% Nulls
GR	60	23	0.383
IR	61	19	0.311
OR	59	24	0.407
OBP	51	10	0.196
Large_Protein_Families	1060	-	-

CNVs (all - not restricted to homozygous calls)			
Family	# Proteins	Nulls >10%	% Nulls
GR	60	17	0.283
IR	61	8	0.131
OR	59	13	0.220
OBP	51	4	0.078
Large_Protein_Families	1060	-	-

Supplementary Table 5 | Summary of SNP, small indel, and CNV protein-disruptive mutations.

Summaries are provided for the chemosensory families and the pooled non-chemosensory families. For each mutation class there are two tables, one for the total data set of disruptive mutations and one conditioning on the disruptive mutations that are at >10% frequency ("Nulls >10%"). The summaries are shown by providing their raw counts ("Nulls") and as the percentage of their family size ("% Nulls"). Red text within the "Nulls" and "Nulls >10%" columns indicate that there are genes harboring multiple independent disruptive mutations.

Mutation	Line	Gene	Expect Mutation?	Support Expectation?	Comments	left primer	right primer
SNP	B51	*Ir60d*	no	yes		GCGAAGATGCAGTGAAGTTG	TTTCCACCAGCTTTCGTTTT
SNP	N10	*Ir60d*	yes	yes		GCGAAGATGCAGTGAAGTTG	TTTCCACCAGCTTTCGTTTT
SNP	T14A	*Or65b*	yes	no	**False Negative:** expected tCa/tGa, but there were 2 SNPs present that generated a tGC (S)	CTTGGTGTCCTTGTCGGTTT	TGTCAGGATTCCATAACATTGG
SNP	I02	*Or65b*	yes	no	**False Negative:** expected tCa/tGa, but there were 2 SNPs present that generated a tGC (S)	CTTGGTGTCCTTGTCGGTTT	TGTCAGGATTCCATAACATTGG
SNP	B04	*Ir54a*	no	yes		GGAAAGAGAGCACCCTACCTC	AAGTCTCCCATCGAAATCCTC
SNP	ZH42	*Ir54a*	yes	no	**False Positive:** SNP was not found in this line	GGAAAGAGAGCACCCTACCTC	AAGTCTCCCATCGAAATCCTC
SNP	T10	*Ir60e*	yes	yes		ATCAAAATGAAGGCGGACAC	ACCCGCTAACCAACTTCCTT
SNP	T25A	*Ir60e*	yes	yes		ATCAAAATGAAGGCGGACAC	ACCCGCTAACCAACTTCCTT
SNP	T22A	*Ir75b*	no	yes		CCTATGTTACTCATGATCCTTTCTC	GTGGTAAAGCGGTCACCTGT
SNP	N25	*Ir75b*	yes	yes		CCTATGTTACTCATGATCCTTTCTC	GTGGTAAAGCGGTCACCTGT
SNP	T04	*Gr36a*	yes	yes		TCAGGTCGCGAAACTCATTA	GCACCTCTGTCTTGAGCAAA
SNP	T14A	*Gr36a*	yes	yes		TCAGGTCGCGAAACTCATTA	GCACCTCTGTCTTGAGCAAA
SNP	T09	*Or35a*	yes	yes		ATTGCACGCATGGGAGACT	CAAGGACATCGCAACAGCTA
Indel	N07	*Or65a*	yes	yes		TCGAAACTGGGATCCGAACA	ACCGATGGATTCACTGATACCA
Indel	N25	*Or65a*	yes	yes		TCGAAACTGGGATCCGAACA	ACCGATGGATTCACTGATACCA
Indel	N10	*IR76a*	yes	yes		TCCTTTTGTTCAGCATGGACA	AGTCCAATCGTTGTAGGGGA
Indel	B05	*Or85a*	yes	yes		TAGTGTTGTTTATGCGGCGG	TTTCCACATCAGTACGCAGC
Indel	B38	*Or85a*	yes	yes		TAGTGTTGTTTATGCGGCGG	TTTCCACATCAGTACGCAGC
Indel	T10	*Ir92a*	yes	yes		cGCGTGCTGTTTCGATAAGA	AAAGGCCAGGACGAAGAGG
Indel	ZH26	*Ir92a*	yes	yes		cGCGTGCTGTTTCGATAAGA	AAAGGCCAGGACGAAGAGG

Supplementary Table 6 | Summary of PCR-based validation of a subset of putative protein-disrupting SNP and indel mutations, and primer sequences.

Supplementary References

1. Mi, H., Muruganujan, A. & Thomas, P. D. PANTHER in 2013: modeling the evolution of gene function, and other gene attributes, in the context of phylogenetic trees. *Nucleic Acids Res* **41**, D377–86 (2013).

2. Galizia, C. G., Münch, D., Strauch, M., Nissler, A. & Ma, S. Integrating heterogeneous odor response data into a common response model: A door to the complete olfactome. *Chem Senses* **35**, 551–63 (2010).

3. Grenier, J. K. et al. Global diversity lines - a five-continent reference panel of sequenced *Drosophila melanogaster* strains. *G3* **5,** 593–603 (2015).

4. Vosshall, L. B. & Stocker, R. F. Molecular architecture of smell and taste in drosophila. *Annual Review of Neuroscience* **30**, 505–533 (2007).

5. Rytz, R., Croset, V. & Benton, R. Ionotropic Receptors (IRs): Chemosensory ionotropic glutamate receptors in *Drosophila* and beyond. *Insect Biochemistry and Molecular Biology* **43**, 888–897 (2013).

6. Freeman, E. G. & Dahanukar, A. Molecular neurobiology of *Drosophila* taste. *Current Opinion in Neurobiology* **34**, 140–148 (2015).

7. Mansourian, S. & Stensmyr, M. C. The chemical ecology of the fly. *Current Opinion in Neurobiology* **34**, 95–102 (2015).
