# Supplementary material for: Extensive local adaptation within the chemosensory system following Drosophila melanogaster's global expansion
Source: Nat Commun. 2016 Jun 13;7:ncomms11855. doi: 10.1038/ncomms11855 (PMC4910016; doi:10.1038/ncomms11855)
Supplement: Supplementary Dataset 7 — Commands for Fst and Fay and Wu's H simulations. [file ncomms11855-s8.docx]

**Supplementary Data File 7 |** Commands for *F_st_* and Fay and Wu’s *H* simulations.

**For *F_st_* simulations:**

Model 1: Migration from branch 5 to 4:

./msms -ms 84 10000 -s 1 -I 5 15 19 19 18 13 -en 0 1 1.325628388 -en 0 2 4.533534852 -en 0 3 3.774153467 -en 0 4 4.650204436 -en 0 5 14.05549979 -ej 0.070277508 2 4 -ej 0.070277508 3 4 -en 0.070277508 4 5.048910008 -ej 0.245935848 1 4 -en 0.245935848 4 5.048910008 -em 0.245935848 5 4 54.80290899 -ej 0.841033067 4 5 -en 0.841033067 5 1 -stat 1 -Fst 2 -stat 1 -Fst 3 -stat 1 -Fst 4 -stat 1 -Fst 5 -stat 2 -Fst 3 -stat 2 -Fst 4 -stat 2 -Fst 5 -stat 3 -Fst 4 -stat 3 -Fst 5 -stat 4 -Fst 5 -b

Model 2: Migration from branch 4 to 5:

./msms -ms 84 10000 -s 1 -I 5 15 19 19 18 13 -en 0 1 1.222669218 -en 0 2 4.702842694 -en 0 3 3.946541085 -en 0 4 4.764590291 -en 0 5 8.963836046 -ej 0.071018081 2 4 -ej 0.071018081 3 4 -en 0.071018081 4 2.039615559 -ej 0.255614255 1 4 -en 0.255614255 4 4.415517896 -em 0.255614255 4 5 53.97506381 -ej 0.810138777 4 5 -en 0.810138777 5 1 -stat 1 -Fst 2 -stat 1 -Fst 3 -stat 1 -Fst 4 -stat 1 -Fst 5 -stat 2 -Fst 3 -stat 2 -Fst 4 -stat 2 -Fst 5 -stat 3 -Fst 4 -stat 3 -Fst 5 -stat 4 -Fst 5 –b

Model 3: Symmetric migration between braches 4 and 5:

./msms -ms 84 10000 -s 1 -I 5 15 19 19 18 13 -en 0 1 1.32839145 -en 0 2 4.674655535 -en 0 3 3.928593489 -en 0 4 4.793899528 -en 0 5 12.44990158 -ej 0.071339124 2 4 -ej 0.071339124 3 4 -en 0.071339124 4 2.151008425 -ej 0.249859236 1 4 -en 0.249859236 4 3.062216224 -em 0.249859236 4 5 50.96231033 -em 0.249859236 5 4 50.96231033 -ej 0.818908093 4 5 -en 0.818908093 5 1 -stat 1 -Fst 2 -stat 1 -Fst 3 -stat 1 -Fst 4 -stat 1 -Fst 5 -stat 2 -Fst 3 -stat 2 -Fst 4 -stat 2 -Fst 5 -stat 3 -Fst 4 -stat 3 -Fst 5 -stat 4 -Fst 5 –b

**For *H* simulations:**

Model 1: Migration from branch 5 to 4:

ms 84 10000 -s 7 -r 1 600 -I 5 15 19 19 18 13 -en 0 1 1.325628388 -en 0 2 4.533534852 -en 0 3 3.774153467 -en 0 4 4.650204436 -en 0 5 14.05549979 -ej 0.070277508 2 4 -ej 0.070277508 3 4 -en 0.070277508 4 5.048910008 -ej 0.245935848 1 4 -en 0.245935848 4 5.048910008 -em 0.245935848 5 4 54.80290899 -ej 0.841033067 4 5 -en 0.841033067 5 1 | sample_stats | cut -f 10 | stats .025 .05 .5 .95 .975

Model 2: Migration from branch 4 to 5:

ms 84 10000 -s 7 -r 1 600 -I 5 15 19 19 18 13 -en 0 1 1.222669218 -en 0 2 4.702842694 -en 0 3 3.946541085 -en 0 4 4.764590291 -en 0 5 8.963836046 -ej 0.071018081 2 4 -ej 0.071018081 3 4 -en 0.071018081 4 2.039615559 -ej 0.255614255 1 4 -en 0.255614255 4 4.415517896 -em 0.255614255 4 5 53.97506381 -ej 0.810138777 4 5 -en 0.810138777 5 1 | sample_stats | cut -f 10 | stats .025 .05 .5 .95 .975

Model 3: Symmetric migration between braches 4 and 5:

ms 84 10000 -s 7 -r 1 600 -I 5 15 19 19 18 13 -en 0 1 1.32839145 -en 0 2 4.674655535 -en 0 3 3.928593489 -en 0 4 4.793899528 -en 0 5 12.44990158 -ej 0.071339124 2 4 -ej 0.071339124 3 4 -en 0.071339124 4 2.151008425 -ej 0.249859236 1 4 -en 0.249859236 4 3.062216224 -em 0.249859236 4 5 50.96231033 -em 0.249859236 5 4 50.96231033 -ej 0.818908093 4 5 -en 0.818908093 5 1 | sample_stats | cut -f 10 | stats .025 .05 .5 .95 .975
